# Amphiphilic Polyoxazoline Copolymer–Imidazole Complexes as Tailorable Thermal Latent Curing Agents for One-Component Epoxy Resins

**DOI:** 10.1021/acsomega.3c07177

**Published:** 2023-11-30

**Authors:** Taha Behroozi Kohlan, Asu Ece Atespare, Mehmet Yildiz, Yusuf Ziya Menceloglu, Serkan Unal, Bekir Dizman

**Affiliations:** † Integrated Manufacturing Technologies Research and Application Center & Composite Technologies Center of Excellence, Sabanci University, Istanbul 34956, Turkey; ‡ Faculty of Engineering and Natural Sciences, Materials Science and Nano Engineering, Sabanci University, Istanbul 34956, Turkey

## Abstract

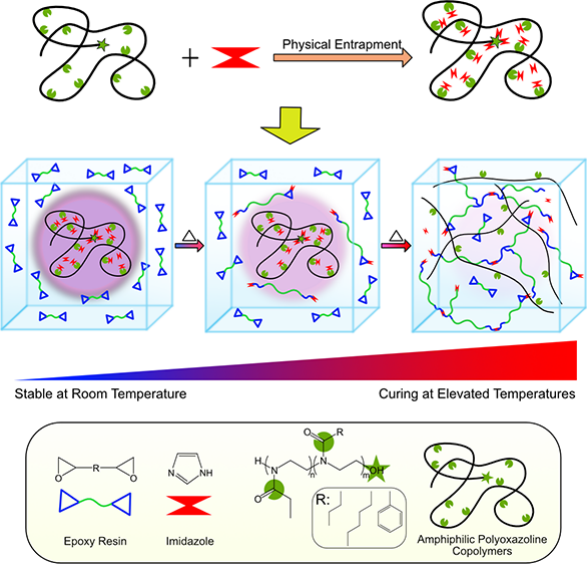

One-component epoxy resins (OCERs) are proposed to overcome the energy inefficiency and processing difficulties of conventional two-component epoxy resins by employing latent curing agents, specifically thermal latent curing agents (TLCs). Despite recent progress, the need for TLCs with a simple preparation method for different curing agents, epoxy resins, and process conditions remains. Here, tailorable TLCs were prepared by forming complexes between imidazole (Im) and amphiphilic polyoxazoline copolymers with tunable structures and properties by a solvent evaporation method. The obtained TLCs were manually mixed with DGEBA to prepare OCERs. The miscibility of the complexes with DGEBA was studied, considering the functionalities of copolymers. The curing behaviors of TLCs were compared using dynamic Differential Scanning Calorimetry (DSC) studies considering the side chain and composition of the copolymers, copolymer:Im ratio, and concentration of Im in DGEBA. The curing behavior of the promising OCERs was studied by isothermal DSC studies to investigate their stability at different temperatures and curing rate at elevated temperatures revealing the stability of these OCERs.

## 1 Introduction

**Scheme 1:**
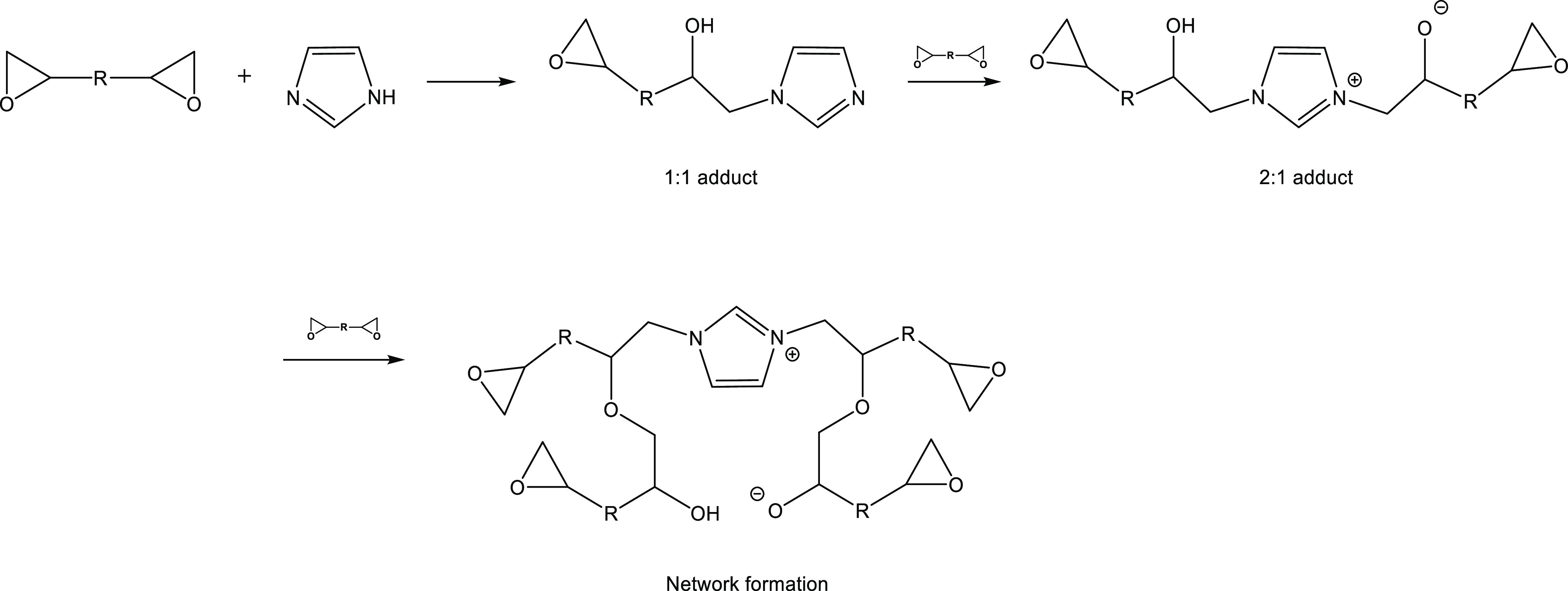
Network Formation and Curing of Epoxy Resins with Im.

Thermoset resins form cross-linked polymer networks when cured.^1^ Among the variety of thermoset resins, epoxy resins are prominent with their utilization in numerous fields including composites,^2^ coatings,^3^ adhesives,^4^ electronics,^5^ etc. due to their favorable mechanical properties, chemical and heat resistance, and low shrinkage upon curing.^6^ Owing to the nature of the oxirane ring having highly polar C–O bonds, numerous curing agents such as amines, anhydrides, hydroxyls, and isocyanates have been reported for curing epoxy resins.^7^ Amines are the most widely used curing agents where the curing of epoxy resins can proceed through step-growth polymerization with primary and secondary amines and through chain-growth polymerization with tertiary amines.^8^ Imidazole (Im) derivatives have attracted a lot of attention due to their ability to provide concurrent curing of epoxy resins through both anionic and base-catalyzed polymerization mechanisms (Scheme 1). Moreover, networks prepared by curing epoxy resins with Im exhibit high heat resistance and modulus and a wide temperature range for curing.^9−11^ However, implementation of Im derivatives in the conventional two-component systems is not desired due to the high reactivity of imidazoles in curing epoxy resins, resulting in the initiation of curing reaction at room temperature.^12^

Major drawbacks for two-component epoxy resins are energy inefficiency, unintended initiation of the curing, increasing viscosity of the prepared mixture over time, a limited process window, unautomated manufacturing methods, and possible human errors in preparing separate batches of the mixture. The mentioned drawbacks lead to processes with inconsistencies in the prepared batches of the mixture that result in nonuniform parts and deviations in properties. Moreover, when mixed, the mixture must be stored and transferred at low temperatures, which imposes an energy and cost burden on the process. To overcome the mentioned drawbacks of two-component epoxy resins, one-component epoxy resins (OCERs) were proposed. In OCERs, the mixture is inert and stable for a long time at ambient conditions and a rapid curing reaction is initiated on demand and in response to a stimulus such as heat and radiation.^13−15^ Among latent curing agents, thermal latent curing agents (TLCs) are mostly investigated in which the curing reaction is initiated at elevated temperatures. Generally, two strategies are used to develop TLCs: (1) using crystalline agents that melt and become active at high temperatures and (2) using conjugates and complexes for encapsulation of curing agents.^16,17^ Dicyandiamide (DICY), an example of crystalline TLCs, melts at around 170 °C and initiates the epoxy curing reaction.^18,19^ Despite the latency provided by DICY, the problem with energy inefficiency remains since high temperatures are required for its processing.^17^ Another method for developing TLCs is based on the formation of complexes/conjugates and encapsulation of conventional curing agents to increase the shelf life of the mixture. For this method, conjugation with small molecules and polymers has been reported. Xu et al. reported conjugating Im with diphenoxychlorophosphine oxide and diphenylphosphinyl chloride to obtain 1-(diphenylphosphinyl)-1*H*-imidazole oxide (DPPIO) and diphenyl 1*H*-imidazol-1-ylphosphonate (DPIPP) as latent curing and flame-retardant agents. The curing studies revealed that the mixtures were stable near room temperature and formed a gel in 6.5 min at 150 °C using 15 wt % of the curing agents.^20^ Despite the latency provided by these systems, the dynamic DSC thermograms revealed the presence of two distinct peaks having peak temperatures of 128.7 and 247.3 °C for DPIPP/EP (7.5 wt %) and 134.6 and 195.2 °C for DPIPP (15 wt %) indicating a wide temperature range for curing. A drawback of the prepared TLC systems is their solid and powder form limiting their miscibility with epoxy resins leading to heterogeneous dispersion and curing. As another strategy, intramolecular hydrogen bonding to prepare TLCs has been reported to be effective. Kudo et al. reported the preparation of 2-(2-hydroxyphenyl)imidazole with hydrogen bonding between the phenolic hydroxyl group and the nitrogen atom of the imidazole ring, which reduced the activity of imidazole toward epoxy resin at room temperature and exhibited rapid curing at 150 °C.^21^ However, a three-roll mill was used to disperse the TLCs in epoxy resin. Since the miscibility of the prepared TLCs plays an important role in the uniformity of the cured part and the feasibility of the process, many attempts have been reported to prepare miscible or soluble TLCs using polymers. Polyethylene glycol (PEG)-based systems with different molecular weights were used to form TLCs by forming conjugates^22^ and complexes^23^ with Im. When complexes were formed, imidazolium cations were formed through interactions between terminal hydroxyl groups of PEG and the pyridine nitrogen of Im, and the polymer matrix was used to wrap around and entrap the Im. Thermal latency in curing was provided by the formed complexes; however, PEG does not offer any other functionality or side chains to tailor the entrapment of Im depending on the desired resin and its functionality or favored curing temperatures. Thus, we sought to implement amphiphilic poly(2-oxazoline) (POZ) copolymers with different side chains to prepare TLCs with Im.

Owing to their unique properties such as controlled synthesis, tunability of properties, versatility, and stimuli-responsiveness, POZs have attracted attention for applications in different fields with a major focus on biomedical applications.^24−27^ 2-oxazoline monomers carrying a variety of functionalities such as aryl or alkyl group can be synthesized through developed methods and later polymerized through cationic ring-opening polymerization (CROP).^28−30^ The versatility of 2-oxazoline monomers paves the way to obtain polymers with desired chemical, physical, and mechanical properties.^31^ Moreover, functionalities of the obtained polymers can be tuned by implementing proper initiating and terminating groups.^32^ However, altering the side chains is the most prevalent method for fine-tuning the polymer properties due to the wide range of monomer choices and the high number of repeating units. Owing to the living nature of CROP, 2-oxazoline monomers can be copolymerized with high control to the desired compositions.^33^ The final properties of copolymers such as amphiphilicity, stimuli-responsiveness, and thermal properties including glass transition temperature (*T*_g_) and crystallinity can be tailored by incorporation of different functionalities through copolymerization of 2-oxazoline monomers.^34^

**Scheme 2:**
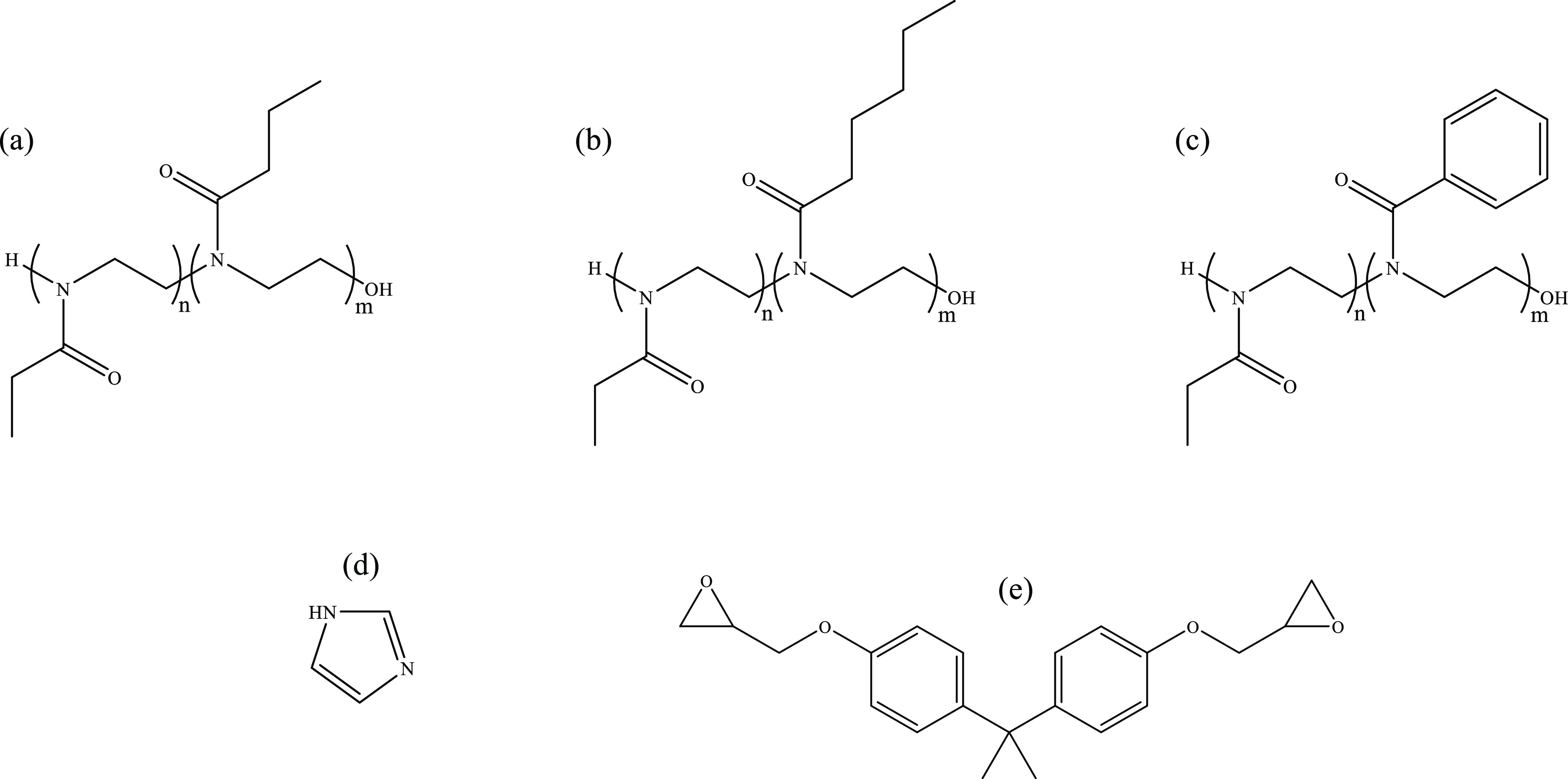
Structure of the Copolymers, Imidazole, and DGEBA Used in This Work: (a) PEOZ–PPrOZ 1K, (b) PEOZ–PPeOZ 1K, and (c) PEOZ–PPhOZ 1K. The composition of the copolymers is shown with *n*:*m* ratios that are 25:75, 50:50, and 75:25; (d) Imidazole; (e) Bisphenol A diglycidyl ether (DGEBA).

Considering the reported studies on the preparation of TLCs, the need for preparing TLCs with tunable curing performance, tailorable structure for high miscibility with the curing agents and resins, and relatively simple and easy-to-prepare methods remains. In this work, amphiphilic POZ copolymers were combined with Im to prepare novel TLCs through forming complexes and entrapment of Im in the copolymer matrix by the solvent evaporation method. The terminal hydroxyl group of POZ copolymers (shown in Scheme 2) was used to form a complex with Im while the copolymer matrices with different functionalities were used to entrap Im. POZ copolymers were utilized to benefit from the possibility of having different alkyl and aryl side chains and compositions on the copolymer matrices to prepare tailorable TLCs for OCERs. OCERs were prepared by manually mixing TLCs with DGEBA. The miscibility of the TLCs and epoxy resin was studied by optical microscopy. The thermal latency behavior of the systems was assessed by dynamic DSC studies considering the Im:DGEBA ratio, copolymer:Im ratio, copolymer composition, and functionality of the side chain as influential parameters. Later, isothermal DSC studies were conducted to examine the stability and curing rate of the OCERs prepared with the promising TLCs.

## 2 Results and Discussion

TLCs were prepared by using POZ-based random copolymers with a molar mass of 1 kg mol^–1^ and Im as the curing agent. The utilized copolymers were prepared by copolymerization of 2-ethyl-2-oxazoline (EOZ) with either one of 2-propyl-2-oxazoline (PrOZ), 2-pentyl-2-oxazoline (PeOZ), or 2-phenyl-2-oxazoline (PhOZ) at three compositions of 25:75, 50:50, and 75:25 with the first number indicating the molar content of poly(2-ethyl-2-oxazoline) (PEOZ) in the copolymer. Complexes of copolymers and Im were formed at 1:1 and 5:1 copolymer: Im ratios. Prepared complexes were mixed with DGEBA to have Im:DGEBA ratios of 1, 3, and 5 wt %. Important curing parameters, namely, normalized enthalpy of curing, the onset temperatures of the first and second peaks, and the main peak temperatures were compared and studied to evaluate the thermal latency behavior of the prepared systems. Moreover, conversion curves were presented for all the curing experiments that show the progress of the curing reaction considering the final value of the enthalpy of curing. The efficiency and performance of the prepared POZ-based TLCs were compared to the curing with pure Im as the reference. Moreover, among the TLCs, a comparison was performed considering the Im content, the copolymer:Im ratio (i.e., 1:1 and 5:1), the composition of the copolymers (i.e., 25:75, 50:50, and 75:25), and the type of side chain present along with 2-ethyl (i.e., 2-propyl, 2-pentyl, and 2-phenyl).

### 2.1 Characterization of Copolymer–Im Complexes

**Figure 1:**
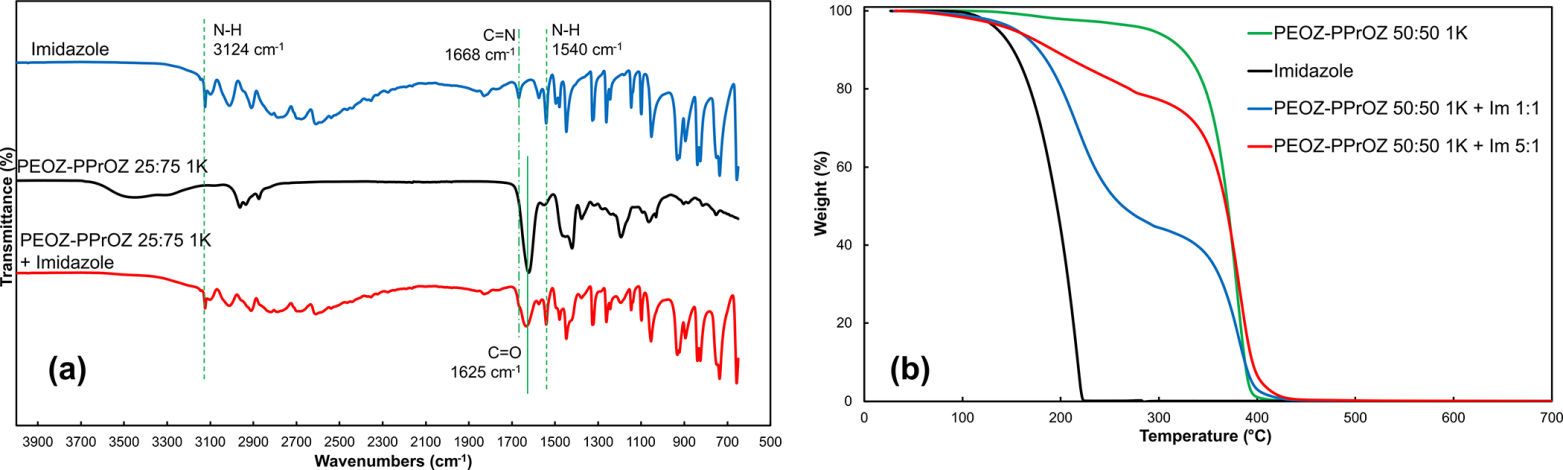
FTIR spectra of Im, PEOZ–PPrOZ 25:75 1K, and their complex (PEOZ–PPrOZ 25:75 1K + Im 1:1) showing the appearance of the imidazole peaks in the copolymer-imidazole complex (a) and TGA thermograms of Im, PEOZ–PPrOZ 50:50 1K, and 1:1 and 5:1 complexes validating the presence of imidazole in the complexes at the intended ratios (b).

To verify the formation of complexes between copolymers and Im, an FTIR spectrum of each complex was recorded. As shown in Figure 1a, characteristic peaks of Im and the copolymer are observed on the spectrum. The sharp C=N peak of Im at 1,668 cm^–1^ is merged with the broader peak of C=O of the copolymer at 1,625 cm^–1^. Moreover, the bending and stretching peaks of N–H of Im are observed at 1,540 and 3,124 cm^–1^, respectively. All of the other peaks of Im are observed in the spectrum of the copolymer–Im complex. Figure 1b shows the TGA thermograms of Im, complexes of copolymer, and Im (i.e., PEOZ–PPrOZ 50:50 1K + Im 1:1 and PEOZ–PPrOZ 50:50 1K + Im 5:1), and PEOZ–PPrOZ 50:50 1K. TGA thermogram of Im shows a single-step rapid degradation starting at 178 °C and ending at 222 °C with less than 0.1 wt % residue. However, when forming complexes with copolymers, the degradation range of Im was widened to start at 172 °C and end at 251.78 °C (276.76 °C) for the 1:1 (5:1) system, indicating the higher thermal stability of Im in complexes. Moreover, the copolymer:Im ratios of 1:1 and 5:1 were verified by the distinctive degradation temperatures of Im and copolymers.

### 2.2 Optical Microscopy Results

**Figure 2:**
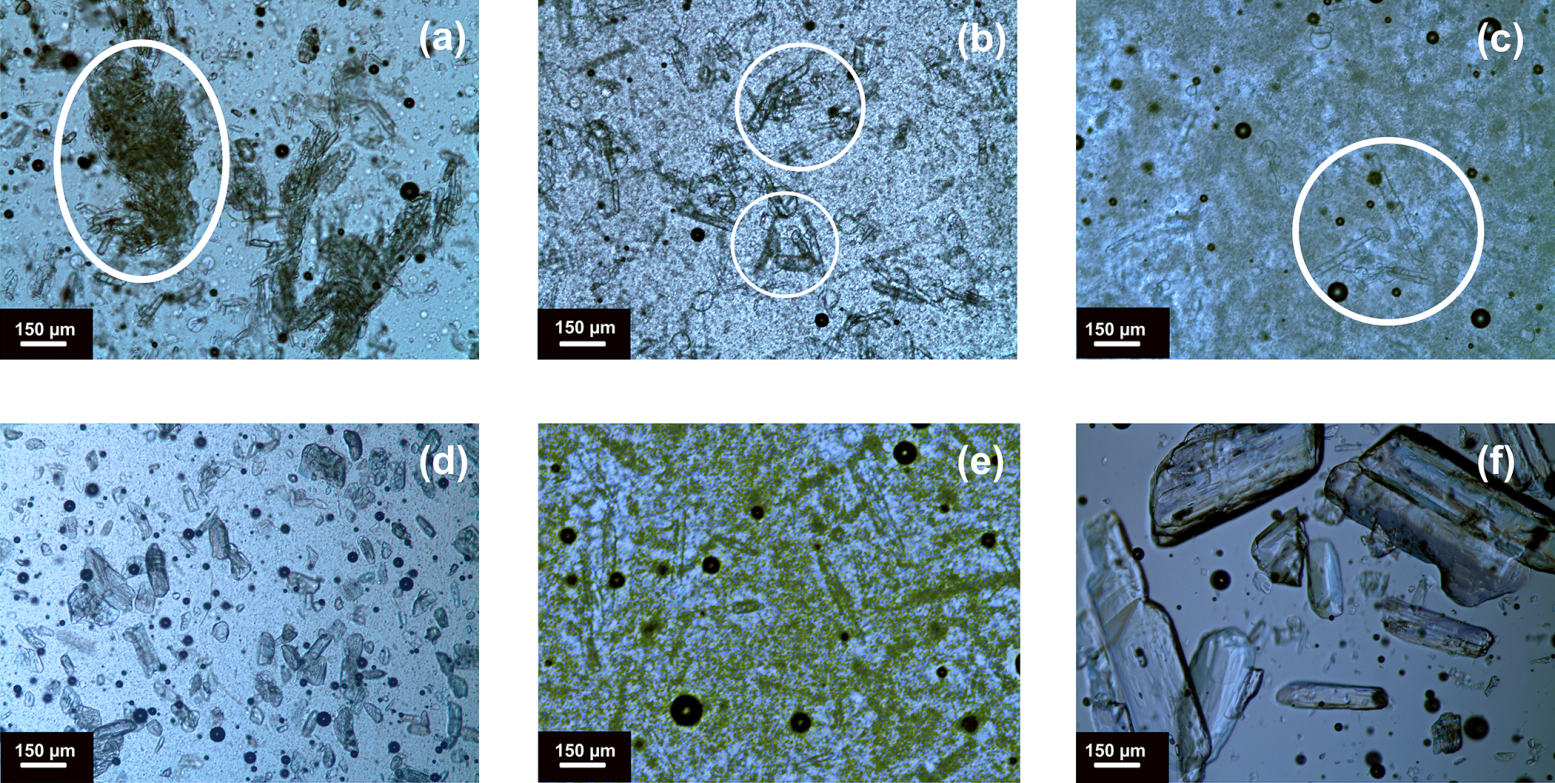
Optical microscopy images of DGEBA mixtures at 5 wt % with PEOZ–PPhOZ 25:75 + Im (a), PEOZ–PPhOZ 50:50 + Im (b), PEOZ–PPhOZ 75:25 + Im (c), PEOZ–PPeOZ 25:75 + Im (d), PEOZ–PPrOZ 25:75 + Im (e), and pure Im (f).

With the changes in the structure of the copolymer matrices within the copolymer library of this study, differences in their miscibility and compatibility with DGEBA were expected. To investigate the dispersion quality and compatibility of the TLCs with DGEBA, optical microscopy images were obtained from the samples prepared by mixing the TLCs and DGEBA at 5 wt % Im content. Figure 2a–c shows the dispersion of PEOZ–PPhOZ-based TLCs with the composition of 25:75, 50:50, and 75:25, respectively.

As can be seen from Figure 2, a decrease in the content of the 2-phenyl substituent of the copolymer has led to the increase in the compatibility and dispersion quality of the TLC with DGEBA as the observed aggregated copolymer–Im complexes in (a) have been replaced by finely dispersed complexes in (c). This behavior demonstrated the incompatibility of the copolymer matrices having a high content of 2-phenyl substituents at this molar mass (1 kg mol^–1^) with DGEBA. Moreover, an increase in the content of 2-ethyl substituent has led to better quality TLC dispersions in DGEBA. Considering the compatibility of the copolymers with high 2-ethyl content, we sought to investigate the compatibility of the copolymers having alkyl side chains at the lowest 2-ethyl content (i.e., 25:75). Comparison of the dispersion quality and compatibility of PEOZ–PPeOZ 25:75 1K and PEOZ–PPrOZ 25:75 1K based TLCs with DGEBA revealed that while the compatibility of these copolymers with DGEBA is relatively similar, PEOZ–PPrOZ based TLCs have smaller aggregate sizes with a more homogeneous dispersion. It can be comprehended that with the observed compatibility of the copolymers of 25:75 composition with DGEBA, 50:50 and 75:25 compositions having higher 2-ethyl content will have better dispersions. Examining the dispersion of pure Im in DGEBA (Figure 2f) revealed that the formation of complexes with copolymers has altered the crystalline structure of Im and lowered the size of aggregates.

Based on the structure of the copolymers and Im, we postulated the working mechanism of the obtained TLCs considering two main contributions of the utilized copolymers. First, complexes were formed between the pyridine nitrogen of Im and the terminal hydroxyl group of the copolymer through hydrogen bonding and protonation of the tertiary amine functionality of Im that has been reported previously by Kudo et al.^21^ The second mechanism is based on the physical entrapment of Im in the copolymer matrix in the entanglements of the copolymer chains that can be obtained by the interactions of the amine functionality of Im and the amide functionality (hydrophilic part) of the copolymers. Subsequently, Im is released from the copolymer matrix at elevated temperatures. It can be postulated that the physically entrapped Im in the copolymer matrix can be released at lower temperatures compared with the Im molecules that have formed a complex with the copolymer chains. Clearly, the type of side chain influences the interactions between the copolymer and Im, the capability of the polymer matrix to entrap Im, and the temperature of its release from the copolymer matrix.

### 2.3 DSC Results of Pure Im

**Figure 3:**
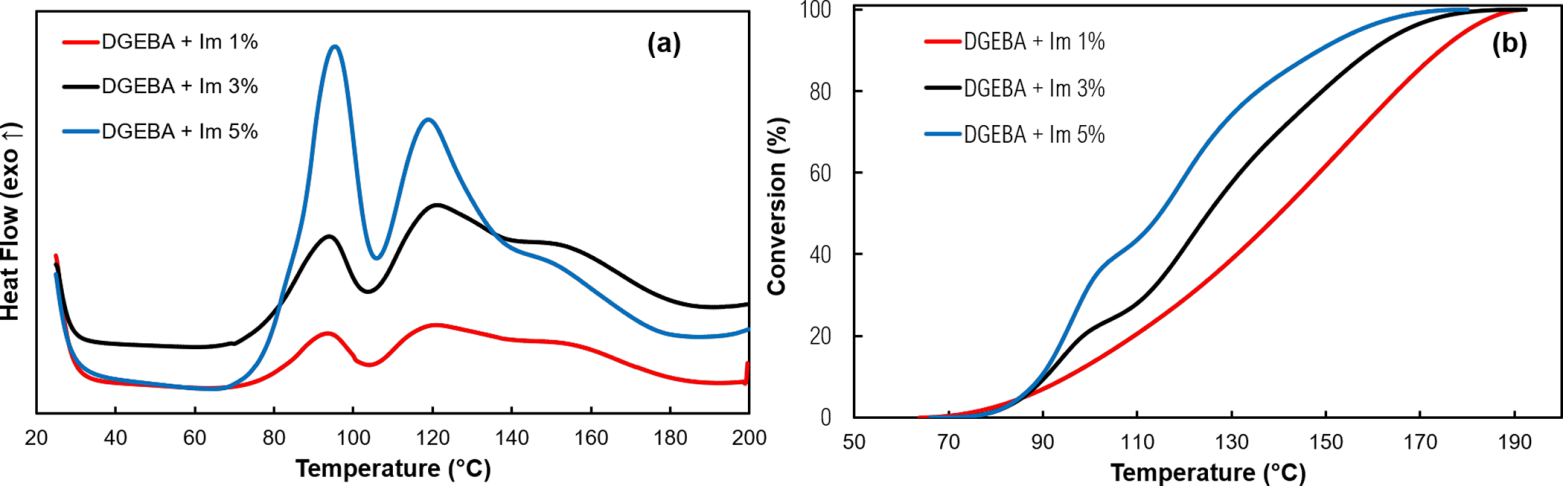
DSC thermograms of curing DGEBA with pure Im (a) and conversion curves (b). Heating rate: 10 °C/min.

DSC thermograms and conversion curves of curing DGEBA with pure Im at 1, 3, and 5 wt % of Im are shown in Figure 3. In agreement with the literature, two distinct peaks are observed on the DSC thermograms.^35^ As shown in Scheme 1 (curing mechanism of DGEBA with Im), the first peak on the thermograms is associated with the first and second adduct formations between the pyridine nitrogen group of Im and the epoxide groups. With the progress of the curing, the second peak appeared at higher temperatures, which is related to the anionic chain growth polymerization of DGEBA. As expected, the 1 wt % sample has the lowest enthalpy among samples due to the lower amount of Im while higher enthalpy values are observed for the 3 and 5 wt % samples. A sharp ascension in conversion curves is observed for 3 and 5 wt % samples, indicating the progression of the adduct formation step when a higher amount of Im is used. Table 1 summarizes the data for curing DGEBA with pure Im. After studying the curing of DGEBA with pure Im, we aimed to study the behavior of the prepared TLCs by first investigating the effects of copolymer:Im ratios at different Im:DGEBA ratios.

**Table 1:** Dynamic DSC Data of Curing DGEBA with Pure Im at Different Im:DGEBA Ratios

Im:DGEBA ratio (wt %)	normalized enthalpy (J/g)	1st peak Onset (°C)	2nd peak onset (°C)	main peak temperature (°C)
1%	55.91	77.95	101.00	93.63
3%	104.22	78.10	101.44	120.90
5%	199.99	78.68	98.00	94.00

### 2.4 DSC Results of PEOZ–PPrOZ 25:75 1:1 and 5:1 1K 1:1 and 5:1

**Figure 4:**
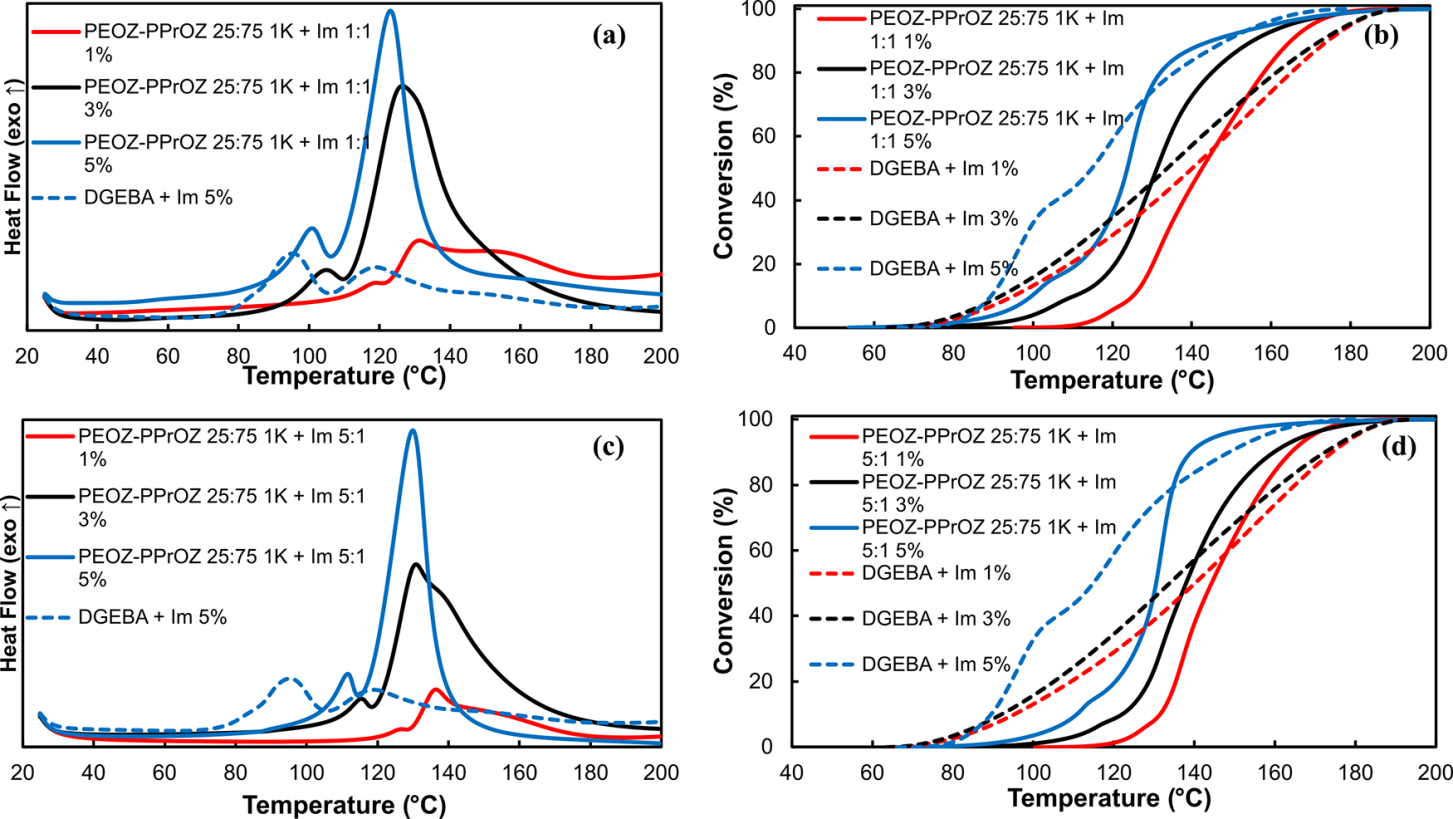
DSC thermograms and conversion curves of curing DGEBA with complexes of Im and PEOZ–PPrOZ 25:75 1K 1:1 (a,b); and 5:1 (c,d). DSC thermogram of curing with pure Im at 5 wt % is shown in dotted lines in (a) and (c). Conversion curves related to curing with pure Im at 1, 3, and 5 wt % are shown as dotted lines in (b) and (d). Heating rate: 10 °C/min.

The effects of increasing copolymer:Im ratio from 1:1 to 5:1 and the effects of Im content on the performance of the TLCs were investigated. The curing behavior of PEOZ–PPrOZ 25:75 1K-based TLCs is presented here, and the data related to the other TLCs are reported in SI (see SI part 2). In contrast to curing with pure Im, using TLC has led to the formation of a curing profile with one major peak at higher temperatures accompanied by a shoulder or a small peak at lower temperatures. This curing profile demonstrated that the thermal latency in curing is provided by all prepared TLCs up to different extents, which was also verified by the increased onset and main peak temperatures and the shift to the higher temperatures in conversion curves compared to the curing with pure Im. Moreover, while having higher onset temperatures for TLCs (Figure 4a,c), higher enthalpy values were also obtained for the TLCs compared to the curing with pure Im at the same Im content. This behavior demonstrated that with the provided latency in curing, the initiation temperature of the curing has increased; thus, anionic chain growth polymerization is the main mechanism of the curing when the prepared TLCs are used. In other words, TLCs have prevented the adduct formation at low temperatures (e.g., 80 °C) and shifted the curing to higher temperatures where the anionic chain growth polymerization occurred, resulting in higher enthalpy values. Furthermore, the introduction of Im to the resin in the form of complexes with the copolymers has increased the solubility of Im in the resin.

Conversion curves of curing with TLCs and pure Im are presented in Figure 4b,d in which the curves related to curing with pure Im are shown as dotted lines. As can be seen, not only have the initiation temperatures of the curing reactions increased with TLCs but also a fast curing behavior was observed for the OCERs at elevated temperatures demonstrated by the sharp increase in the conversion values after initiation of the curing.

Comparing the samples with copolymer:Im ratios of 1:1 and 5:1, higher onset and main peak temperatures were observed for the 5:1 samples indicating a slower release of Im from the copolymer–Im complexes when higher content of copolymer is used (Table 2). This phenomenon agrees with the postulated working mechanism of TLCs that both the formation of complexes with the copolymer and physical entrapment of Im in the copolymer matrix were accounted for. When the 5:1 copolymer:Im ratio was used, a higher number of copolymer chains and hydroxyl end groups were available to form complexes and entrap the Im in the copolymer matrix. This phenomenon has led to the slower release of Im and thus higher onset temperatures for the initiation of the curing reactions. For the TLCs based on PEOZ–PPrOZ 1K and PEOZ–PPeOZ 1K copolymers with different copolymer compositions (i.e., 25:75, 50:50, and 75:25), similar values for the enthalpy of curing were obtained for both 1:1 and 5:1 copolymer:Im ratio. On the other hand, curing with PEOZ–PPhOZ-based TLCs at a 5:1 ratio resulted in lower enthalpy values compared to the 1:1 samples (see Figures S6–S8; and Tables S6–S8 in SI). This behavior is associated with the incompatibility of the PEOZ–PPhOZ copolymer matrix with DGEBA as discussed in part 2.2 and the incompatibility of Im and this copolymer matrix resulting from the hydrophilicity of Im and hydrophobicity of the copolymer matrices having 2-phenyl groups. When PEOZ–PPhOZ 1K + Im 5:1 based TLCs were used for curing, a lower dispersion of the copolymer in the resin and lower content of free Im in the copolymer–Im matrix resulted in incomplete curing and lower enthalpy values.

**Table 2:** Dynamic DSC Data of PEOZ–PPrOZ 25:75 1K-based OCERs at Different Copolymer:Im and Im:DGEBA Ratios

copolymer: Im ratio	Im:DGEBA ratio(wt %)	normalized enthalpy (J/g)	1^st^ peak onset (°C)	2^nd^ peak onset (°C)	main peak temp.(°C)
1:1	1%	144.42	111.25	121.26	131.22
1:1	3%	535.91	94.10	112.25	126.53
1:1	5%	481.99	92.14	109.87	123.17
5:1	1%	116.48	120.79	128.93	136.42
5:1	3%	451.49	109.34	120.58	130.87
5:1	5%	493.14	105.27	117.87	129.98

When the increase in the content of Im was examined, a general trend of an increase in the enthalpy values and a decrease in the onset and main peak temperatures was observed. The decrease in the onset temperatures is also reflected on the conversion curves as the curves related to 5 wt % samples ascended at lower temperatures compared to 3 and 1 wt % samples. Moreover, the first peak of curing was observed more distinctly when the Im content was increased in the curing experiment. This behavior was expected since a higher content of the Im that was physically entrapped in the copolymer matrix was presented with the increase in the amount of the introduced copolymer–Im complexes to the resin. Thus, more of the free Im was available at lower temperatures (e.g., 80 °C) as it was gradually released from the copolymer matrix, initiating the adduct formation at lower temperatures. Furthermore, the first peak of curing was more pronounced for 1:1 samples compared to that for 5:1 samples. This behavior is explained by the postulated working mechanism of the TLCs as for the 5:1 samples a higher content of Im has formed complexes with the terminal group of the copolymer instead of just being physically entrapped in the copolymer matrix. Having a library of copolymers with different compositions and side chains, we observed different TLC behaviors and turned our attention to investigating the effects of the copolymer composition on the TLC behavior of the prepared systems.

### 2.5 DSC Results for the Effect of Composition

**Figure 5:**
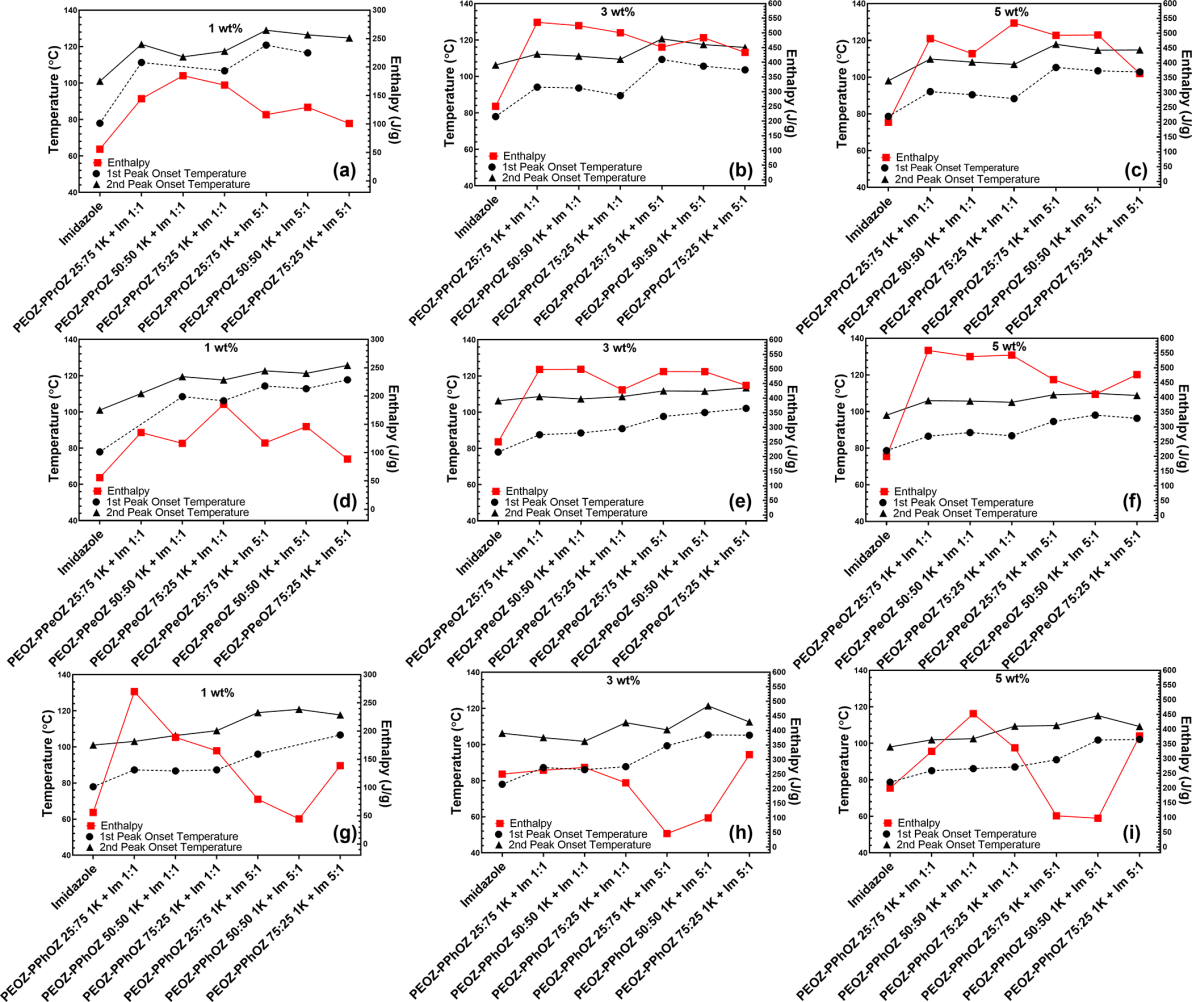
Comparison of the first and second onset temperatures and enthalpy values of TLC systems and pure imidazole based on copolymer composition for PEOZ–PPrOZ 1K (a–c); PEOZ–PPeOZ 1K (d–f); PEOZ–PPhOZ 1K (g–i). With imidazole being the very left data point on each subfigure, an increasing trend in onset temperatures despite the variations in enthalpy is observed as an indicator of the provided latency in curing.

The effects of the copolymer composition on the TLC behavior of the prepared systems were investigated in the second part while keeping the Im:DGEBA ratios constant in each subfigure. Investigating the PEOZ–PPrOZ 1 K-based OCERs (Figure 5a–c), obtained enthalpy values were in the same range at the same copolymer:Im ratio and Im content. For instance, enthalpy values for 1:1 samples at 3 wt % Im content are 500, 524, and 535 J/g for 25:75, 50:50, and 75:25 copolymer compositions, respectively. This behavior demonstrated that the PEOZ–PPrOZ 1K copolymers with different compositions were of similar compatibility with the resin, which resulted in a similar behavior for the release of Im. However, considering the onset and main peak temperatures, TLCs with PEOZ–PPrOZ 25:75 exhibited the highest values for both 1:1 and 5:1 samples, meaning that the increase in the content of PPrOZ in the copolymer resulted in a slower release of Im.

As the next group of copolymer–Im complexes, thermal latency behavior of PEOZ–PPeOZ 1K-based TLCs was investigated (Figure 5d–f) exhibiting similar curing behaviors and profiles (Figures S10 and S13 and Tables S3, S4, and S5). Considering the onset and main peak temperatures, the PEOZ–PPeOZ 75:25 1K-based systems exhibited higher values. This behavior can be related to the glass transition temperature (*T*_g_) of the copolymers. As the content of the 2-pentyl side group was increased in the copolymer, *T*_g_ had a decreasing trend with values of 18 °C and −3 °C for PEOZ–PPeOZ 75:25 1K and PEOZ–PPeOZ 25:75 1K, respectively.^30^ In other words, the low *T*_g_ value and the wax form of the PEOZ–PPeOZ 25:75 1K copolymer have led to the dissolution of the copolymer matrix in DGEBA at lower temperatures and subsequent early release of the entrapped Im compared to the PEOZ–PPeOZ 75:25 1K copolymer. The effects of the miscibility and compatibility of the copolymer with DGEBA are more pronounced in 5:1 samples in which a higher amount of copolymer is introduced to the resin and PEOZ–PPeOZ 75:25 1K-based TLCs, as the copolymer with a higher *T*_g_ value, exhibited better performance.

The curing data of PEOZ–PPhOZ 1K-based TLCs are shown in Figures 5g–i, and the curing profiles are shown in Figures S11 and S14. As can be seen from the curing profiles of 1:1 samples, PEOZ–PPhOZ 75:25 1K-based systems have the lowest intensity for the first peak of curing, while with the increase in the content of 2-phenyl substituent of the copolymer, more pronounced first peaks were observed. With respect to the proposed working mechanism of the TLCs, this phenomenon indicated that the presence of 2-phenyl groups reduced the ability of copolymer in both entrapping Im in the copolymer matrix and formation of complexes between the copolymer functionalities and Im. Additionally, with the increase in the content of 2-phenyl side chains, the hydrophilicity of the copolymer is drastically reduced, thus making the copolymer matrix more incompatible with Im. As a result, a higher amount of free Im was accessible for initiation of the curing reaction at lower temperatures. Furthermore, the electron-withdrawing characteristic of the 2-phenyl group can be considered to be an influential factor in the strength of the formed complexes. The mentioned parameters have resulted in shifting the onset and main peak temperature to lower temperatures with an increase in the 2-phenyl content of the copolymer matrix.

For the enthalpy values of the 5:1 samples, while PEOZ–PPhOZ 75:25 1K-based TLCs exhibited expected values, an increase in the content of the 2-phenyl group resulted in decreased enthalpy values. Moreover, for PEOZ–PPhOZ 50:50 1K and PEOZ–PPhOZ 25:75 1K-based TLCs, an increase in the content of Im in the curing from 1 to 3 and 5 wt % did not increase the enthalpy values with the expected trend observed for the other systems. This phenomenon can be correlated with the incompatibility of these copolymer matrices with Im and the observations discussed in the optical microscopy images in which the presence of 2-phenyl substituents in the copolymer structure decreased the miscibility of the copolymer matrix with resin. Thus, with the increased content of copolymer from 1:1 to 5:1, the lower content of free Im led to lower enthalpy values.

### 2.6 DSC Results for the Effect of Side Chains

**Figure 6:**
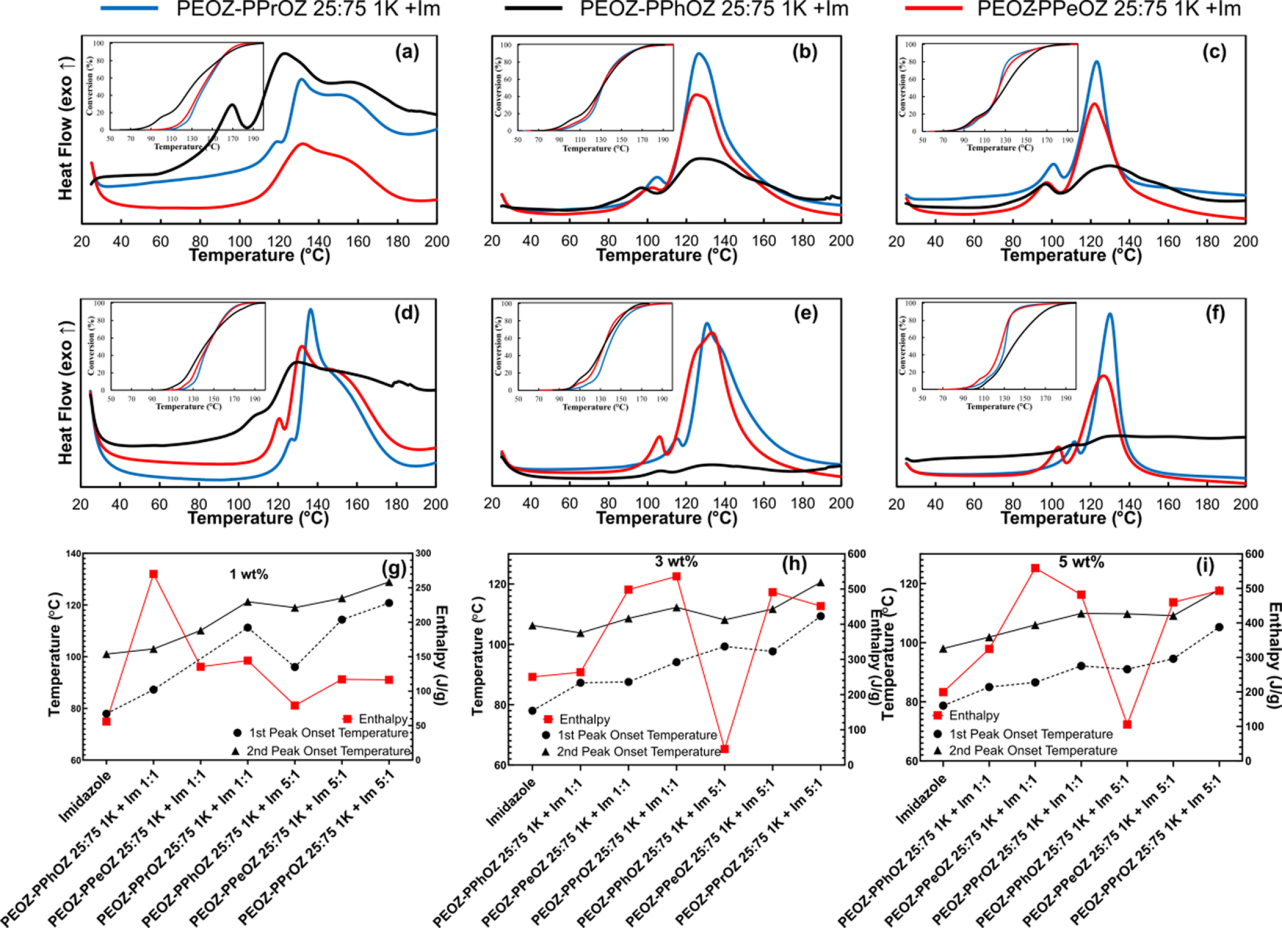
DSC thermograms of curing of DGEBA with copolymers-Im complexes having the composition of 25:75 at 1:1 ratio and 1 wt % (a), 3 wt % (b), and 5 wt % (c); at 5:1 ratio and 1 wt % (d), 3 wt % (e), and 5 wt % (f). Comparison of the first and second onset temperatures and enthalpy values of TLC systems and pure imidazole for 1 wt % (g), 3 wt % (h), and 5 wt % (i) with imidazole being the very left data point in each subfigure an increasing trend in onset temperatures despite the variations in enthalpy is observed as an indicator of the provided latency in curing. Heating rate: 10 °C/min.

With the obtained insight into the effects of the composition of the copolymers on the TLC behaviors, we sought to study the effects of the side chains of the copolymer in these systems. Figure 6 shows data related to curing experiments with copolymers that have a composition of 25:75 as the lowest 2-ethyl content in the copolymers. In Figure 6g–i, data related to the curing with pure Im is also presented as the reference to evaluate the TLC behavior of the samples.

DSC profiles depicted in Figure 6 revealed that PEOZ–PPrOZ 25:75 1K and PEOZ–PPeOZ 25:75 1K-based TLCs exhibited similar behavior, while PEOZ–PPhOZ 25:75 1K-based TLCs showed different behavior. Considering that the copolymers with the composition of 25:75 had the highest content of the alkyl(2-propyl, 2-pentyl) or aryl(2-phenyl) side chain copolymerized with EOZ, the effects of the side chain were more pronounced. For the PEOZ–PPhOZ 25:75 1K-based TLCs, lower onset temperatures along with lower enthalpy values were observed. This behavior indicated the cooccurrence of an early release of Im from the copolymer matrix and the incompatibility of the copolymer matrix with the resin and Im.

Comparing the copolymers having 2-propyl and 2-pentyl side chains, enhanced TLC behavior is observed for PEOZ–PPrOZ 25:75 1K-based TLC. This can be attributed to the higher *T*_g_ value of PEOZ–PPrOZ 25:75 1K compared to PEOZ–PPeOZ 25:75 1K. The higher *T*_g_ has led to better entrapment of Im in the polymer matrix at elevated temperatures.

The ascending trend in the onset temperatures from 1:1 to 5:1 systems confirmed the effect of increasing the copolymer:Im ratio as with the increase in the copolymer content, higher onset temperatures were observed. Furthermore, it can be seen that PEOZ–PPrOZ 25:75 1K-based TLCs have the highest onset temperatures in both 1:1 and 5:1 systems at all Im contents.

**Figure 7:**
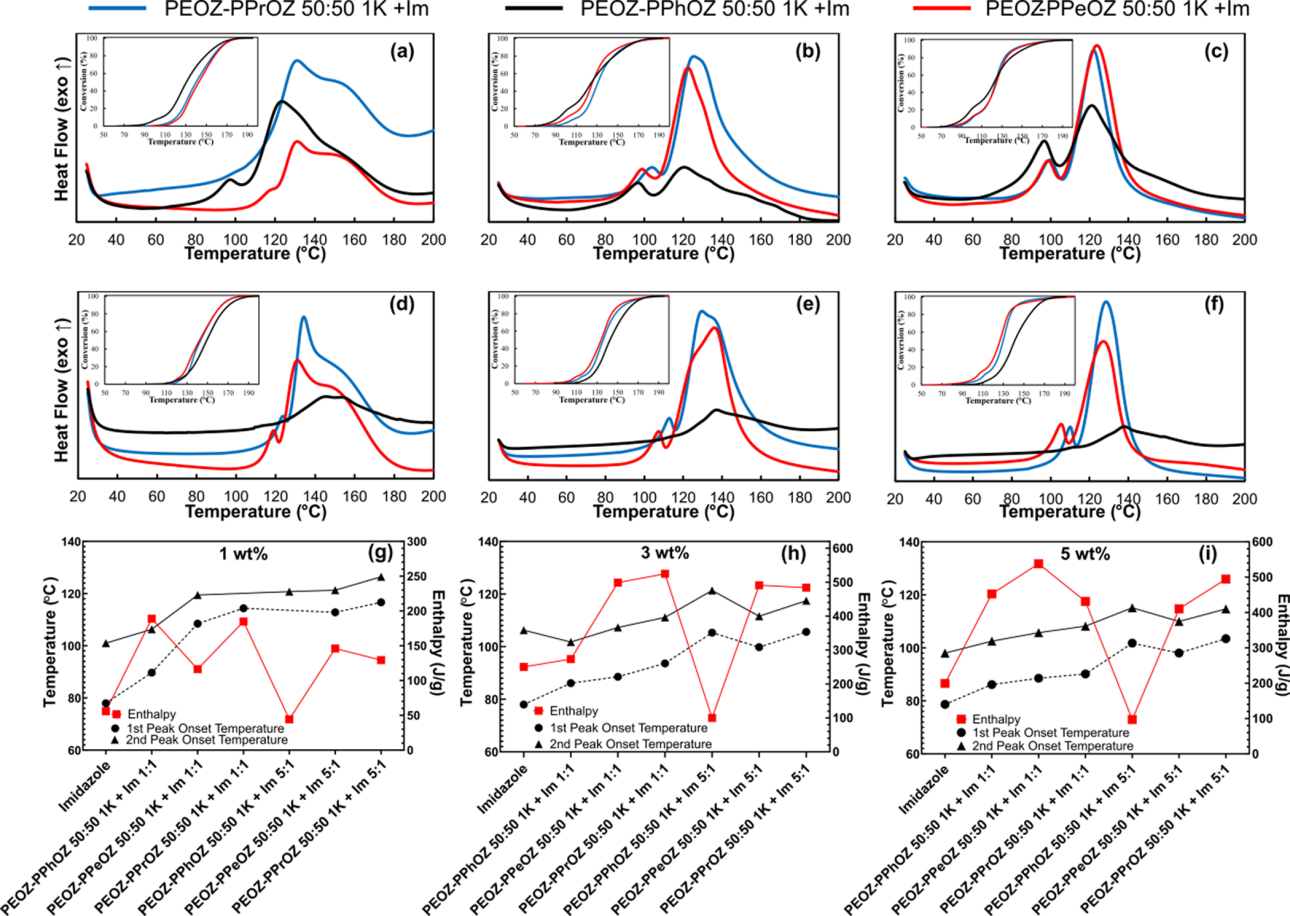
DSC thermograms of curing of DGEBA with copolymers-Im complexes having the composition of 50:50 at 1:1 ratio and 1 wt % (a), 3 wt % (b), and 5 wt % (c); at 5:1 ratio and 1 wt % (d), 3 wt % (e), and 5 wt % (f). Comparison of the first and second onset temperatures and enthalpy values of TLCs and pure Im for 1 wt % (g), 3 wt % (h), and 5 wt % (i) with imidazole being the very left data point in each subfigure an increasing trend in onset temperatures despite the variations in enthalpy is observed as an indicator of the provided latency in curing. Heating rate: 10 °C/min.

Figure 7 shows the curing data for the copolymers with the composition of 50:50 on their TLC behavior. Like the 25:75 systems, PEOZ–PPhOZ 50:50 exhibited a different curing behavior compared to that of the copolymers with alkyl side chains. Considering the copolymers with alkyl side chains, similar behavior is observed with a slightly better thermal latency behavior of the PEOZ–PPrOZ 50:50 1K-based systems arising from the higher *T*_g_ of this copolymer compared to PEOZ–PPeOZ 50:50 1K.

The ascending trend of the onset temperatures in Figure 7g–i indicated the better thermal latency behavior of the system having 2-propyl side chains (i.e., PEOz-PPrOZ 50:50 1K). The comparable enthalpy values for the 1:1 and 5:1 ratios of PEOz-PPrOZ 50:50 1K and PEOZ–PPeOZ 50:50 1K based TLCs revealed the compatibility of these copolymer matrices with DGEBA that led to the satisfactory release of Im from the copolymer matrix.

**Figure 8:**
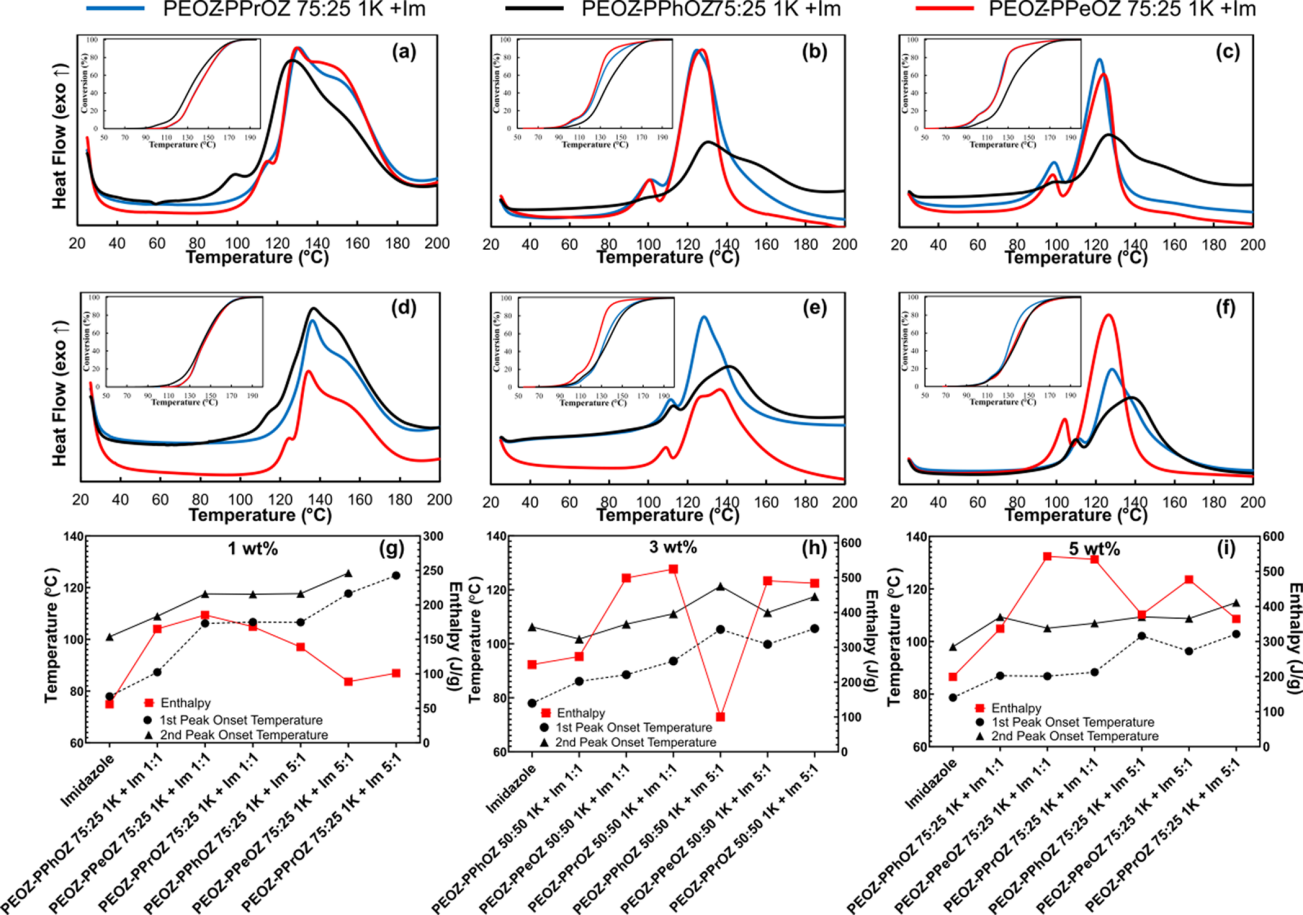
DSC thermograms of curing of DGEBA with copolymers-Im complexes having the composition of 75:25 at 1:1 ratio and 1 wt % (a) 3 wt % (b), 5 wt % (c), at 5:1 ratio and 1 wt % (d), 3 wt % (e), and 5 wt % (f). Comparison of the first and second onset temperatures and enthalpy values of TLCs and pure Im for 1 wt % (g), 3 wt % (h), and 5 wt % (i) with imidazole being the very left data point in each subfigure an increasing trend in onset temperatures despite the variations in enthalpy is observed as an indicator of the provided latency in curing. Heating rate: 10 °C/min.

Finally, the effects of the side chain of the copolymers with the composition 75:25 were investigated by the data shown in Figure 8. Differences in the TLC behaviors arising from having different side chains are minimized for these systems since the copolymers with 75:25 composition have the highest 2-ethyl content which has led to similar performances. As can be seen for the systems having the composition of 75:25, PEOZ–PPhOZ-based systems exhibited similar behavior to the systems with alkyl side chains. However, a lower enthalpy and faster release of Im are still observed for these systems confirming the previous discussions on the incompatibility of these matrices with DGEBA and Im. When the thermal latency behavior of the copolymers with alkyl side chains was examined, similar values for the curing parameters were obtained when used at 1:1 copolymer:Im ratio. However, when introduced at 5:1 ratio, PEOZ–PPrOZ based systems exhibited an enhanced performance.

Concluding the effects of the side chains of the copolymers on the TLC behavior of the prepared systems, it was observed that the presence of 2-phenyl substituents on the copolymer structure reduced the capability of the copolymer matrix to entrap Im. Considering the proposed working principle of the TLCs and obtained optical microscopy images, the incorporation of 2-phenyl groups weakened the interactions between the copolymer and Im and reduced the compatibility of the copolymer matrices with DGEBA. In addition to the weaker interactions leading to faster release of Im, the difference in the hydrophilicity of the Im and PEOZ–PPhOZ copolymers has led to incompatibility of the copolymer matrix and Im leading to higher availability of free Im at lower temperatures that was reflected by the presence of the first peak of curing for these systems. Additionally, the incompatibility of PEOZ–PPhOZ-based systems with DGEBA was verified by having lower enthalpy values when the copolymer:Im ratio was increased to 5:1. Examining the systems based on copolymers with alkyl side chains, similar behavior was observed with a slightly better performance of PEOZ–PPrOZ-based TLCs. This can be attributed to the higher *T*_g_ value of PEOZ–PPrOZ compared to PEOZ–PPeOZ copolymers at different compositions. On the other hand, TLCs based on PEOZ–PPrOZ and PEOZ–PPeOZ exhibited similar behaviors with 1:1 and 5:1 copolymer:Im ratios, indicating the compatibility of these polymer matrices with DGEBA even at high contents. It was observed that numerous parameters such as amphiphilicity and miscibility act as influential factors in determining the curing behaviors of TLCs and that it is necessary to study each case with its comparable systems.

### 2.7 Isothermal DSC Studies

**Figure 9:**
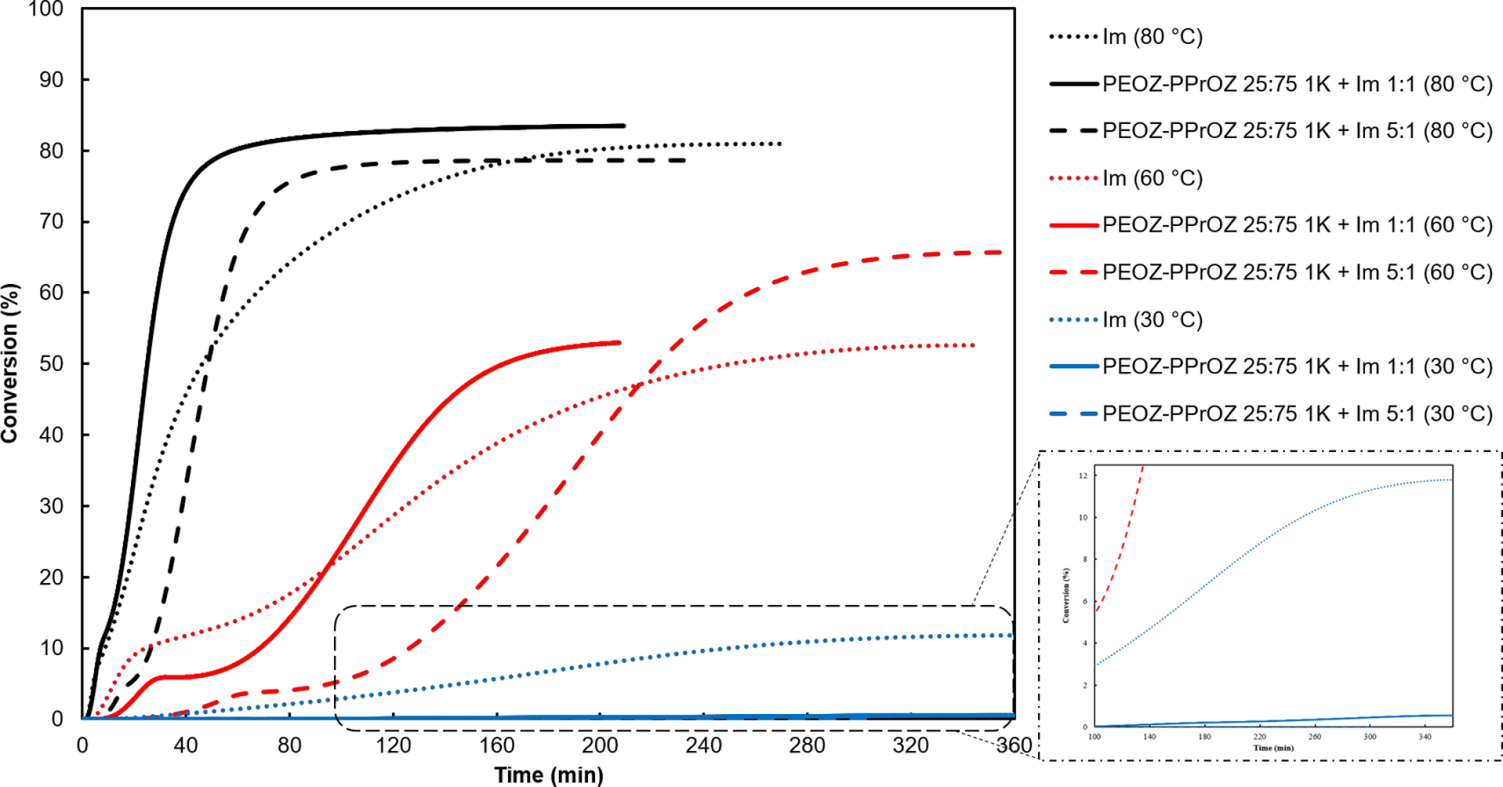
Conversion curves of isothermal DSC studies of prepared one-component epoxy systems at 3 wt % of Im. The dotted lines are related to curing with pure Im.

Isothermal DSC studies were conducted to study the thermal latency behavior of the developed TLCs in one-component epoxy systems that are required to be inert at room temperature and to provide rapid curing at elevated temperatures. Considering the onset and main peak temperatures, 30, 60, and 80 °C were selected to observe the thermal latency and rapid curing behavior at 3 wt % of Im content. As promising systems, PEOZ–PPrOZ 25:75 1:1 and 5:1 TLCs were used to prepare OCERs. Figure 9 shows the conversion values of the prepared one-component resins during the isothermal studies in 6 h considering the total enthalpy of curing obtained from summing the enthalpy of the isothermal run and the enthalpy of the subsequent dynamic run. As shown in Figure 9, PEOZ–PPrOZ 25:75 1K 5:1 shows no curing in the isothermal run and is stable at 30 °C. Moreover, PEOZ–PPrOZ 25:75 1K 1:1 is stable at 30 °C with having 0.5% conversion after 6 h. In addition to thermal latency, investigating the PEOZ–PPrOZ 25:75 1K 1:1 and 5:1 systems at elevated temperatures of 60 and 80 °C demonstrates the rapid curing behavior of these systems compared to curing with pure Im at the same temperature. Furthermore, the provided latency in curing can also be observed at 60 and 80 °C in which OCERs with TLCs have lower conversions compared to the samples with pure imidazole at starting times. To study the long-term stability of the systems, OCERs of PEOZ–PPrOZ 25:75 1K 5:1 and pure Im were stored at room temperature. The residual enthalpy of curing after 3 days of storage is shown in Figure S15. Despite the higher remaining enthalpy for the PEOZ–PPrOZ 25:75 1K 5:1 system, it does not show long-term stability. Possible methods to increase the shelf life will be investigated in our group.

## 3 Conclusions

TLCs based on amphiphilic POZ copolymers and Im were prepared to address the need for tailorable TLCs for epoxy resins. Copolymers with a molar mass of 1 kg mol^–1^ were obtained through copolymerization of EOZ with either PrOZ, PeOZ, or PhOZ at three compositions of 25:75, 50:50, and 75:25. Followed by the successful synthesis of the copolymers, TLCs were obtained by solvent evaporation method at copolymer:Im ratios of 1:1 and 5:1. Miscibility of the prepared systems with DGEBA was studied by optical microscopy. OCERs were prepared by mixing TLCs and DGEBA at Im:DGEBA ratios of 1, 3, and 5 wt %. Thermal latency and curing behavior of OCERs were evaluated by dynamic and isothermal DSC studies. The OCERs based on the copolymers with alkyl groups exhibited a better performance compared to the copolymers having 2-phenyl substituents. It was concluded that the presence of 2-phenyl groups on the copolymer structures reduces the compatibility of the copolymer matrix with Im resulting from their distinct hydrophilicity. Moreover, the *T*_g_ of the copolymers was shown to be influential for the PEOZ–PPrOZ 1K and PEOZ–PPeOZ 1K-based systems as the lower *T*_g_ of the PEOZ–PPeOZ copolymers resulted in a faster release of Im. It was shown from the isothermal studies that PEOZ–PPrOZ 25:75-based OCERs were stable at 30 °C with no curing for 5:1 system and less than 0.5% curing for a 1:1 system in 6 h. Moreover, the rapid curing behavior of the TLC systems after initiation of curing was shown by isothermal DSC studies where a sharp increase in the conversion values was observed for TLCs compared to the gradual increase in curing with pure imidazole. A deeper understanding of the structure–property relationship of the POZ-based polymers and the compatibility and miscibility behavior of these polymers with various curing agents and epoxy resins, and further isothermal DSC studies will help design better TLCs. Furthermore, it should be noted that the effects of polyoxazolines on the thermal, mechanical, and adhesive properties of the manufactured epoxy parts remain to be investigated and are being studied in our group.

## 4 Experimental Section

### 4.1 Materials

EOZ, PhOZ, chlorobenzene, methanol, hexanoic acid, and DGEBA (MW: 340.41 g·mol^–1^, viscosity: 4000–6000 cP at 25 °C) were purchased from Sigma-Aldrich. Trifluoromethanesulfonic acid (TfOH) and oxalyl chloride were purchased from Acros Organics. Butyronitrile and 2-chloroethylamine hydrochloride were purchased from ABCR. Sodium hydroxide (NaOH) and potassium hydroxide (KOH) were purchased from ISOLAB. Ethanolamine, CaH_2,_ dichloromethane (DCM), acetonitrile, zinc acetate dihydrate (Zn (OAc)_2_.2H_2_O), triethylamine (TEA), and sodium sulfate (Na_2_SO_4_) were purchased from Merck. Unless stated otherwise, all chemicals were used as received. Monomers and chlorobenzene were dried over calcium hydride (CaH_2_), distilled, and kept over 4 Å molecular sieves before their use in polymerizations.

### 4.2 Synthesis of Monomers and Copolymers

PrOZ and PeOZ monomers were synthesized following the procedures reported by our group^30^ and synthetic procedures are available in SI (1.1.). Copolymers were synthesized through the copolymerization procedure and conditions reported by our group.^30^ Shortly, polymerizations were conducted in round-bottom flasks dried at 120 °C, cooled under vacuum, and treated with vacuum/N_2_ cycles. 4 M concentration of monomers in chlorobenzene was used for the polymerizations considering the total concentration of monomers in each reaction. TfOH was used as the initiator and termination was done using methanolic KOH overnight. To obtain copolymers with a molar mass of 1000 g/mol, a [M]/[I] ratio of 10 was used. Table 3 summarizes the reaction times, conditions for the polymerizations, and size exclusion chromatography (SEC) results of obtained molar masses and polydispersity (*Đ*) values based on two standards of PMMA and PEOZ. The obtained polymers after purification (for the purification procedure, see SI part 1.2) were kept under N_2_ to prevent moisture absorption. All compositions of each copolymer were synthesized under the same conditions.

**Table 3:** Polymerization Conditions and SEC Results of the Copolymers

		temperature (°C)	time (min)	SEC			
				PEOZ std		PMMA std	
				*M*_p_ (Da)	*Đ*	*M*_p_ (Da)	*Đ*
PEOZ–PPrOZ 1K	25:75	80	30	794	1.18	1594	1.61
	50:50			752	1.15	1206	1.50
	75:25			678	1.18	1305	1.79
PEOZ–PPeOZ 1K	25:75	80	60	1000	1.13	2076	1.20
	50:50			964	1.20	1956	1.25
	75:25			925	1.21	1903	1.58
PEOZ–PPhOZ 1K	25:75	95	60	1189	1.33	2501	1.24
	50:50			1035	1.12	2157	1.14
	75:25			863	1.13	1759	1.15

### 4.3 Preparation of Polymer–Im Complexes

The solvent evaporation method was employed to prepare the copolymer–Im complexes. For PEOZ–PPrOZ 1K and PEOZ–PPeOZ 1K-based TLCs, DCM was used. Based on the desired copolymer:Im ratio, the copolymer was dissolved in DCM to form a 10 wt/v% solution followed by the addition of Im. Then, the solution was heated to reflux for 2 h. When cooled to room temperature, DCM was evaporated under reduced pressure and the obtained copolymer–Im complex was further dried under vacuum overnight and stored under N_2_ to prevent moisture absorption. For PEOZ–PPhOZ 1K-based systems, the same procedure was followed using acetonitrile as the solvent.

### 4.4 Preparation of DGEBA and Copolymer–Im Mixtures

Aiming for the ease of processing and feasibility, the mixing procedure was developed to be as simple as possible, avoiding any solvent or mechanical mixing process. To have uniform mixtures and minimize the effects of mixing, all samples were prepared in 2 mL sample vials. For each sample, approximately 200 mg of DGEBA was used. The mixture was prepared in a way to have Im:DGEBA ratios of 1, 3, and 5 wt %. The copolymer–Im complex was weighed in a dry vial, considering the copolymer:Im ratio of the prepared complexes and the desired Im:DGEBA ratio. Later, DGEBA was added to the vial. The mixture was prepared by slow manual mixing using a spatula to avoid bubble formation for 1 min under ambient conditions. For instance, to prepare a sample with 3 wt % Im:DGEBA ratio using a PEOZ–PPrOZ 25:75 1K-based copolymer–Im complex with 5:1 (1:1) copolymer:Im ratio, 36 mg (12 mg) of the complex was weighed in a vial and 200 mg of DGEBA was added to the vial and mixed for 1 min.

### 4.5 Characterization Methods

Differential scanning calorimetry (DSC) measurements were carried out by using the Mettler Toledo DSC 3+ instrument. For the dynamic DSC experiments, 13–16 mg of the sample was placed in an aluminum pan and experiments were conducted under N_2_ at a heating rate of 10 °C/min. The onset temperature of the curing experiments is the temperature where the tangent line on the curing thermogram intersects with the baseline used for determining the enthalpy of curing. TGA measurements were performed on a Mettler Toledo TGA/DSC 3+ in the range from 25 to 700 °C. Samples of 10–100 mg were heated at 10 °C/min under a nitrogen atmosphere with a flow rate of 100 mL.min^–1^. The onset temperatures of thermal degradation are obtained where the tangent line at the inflection point of the TGA thermogram intersects with the baseline drawn at the point where the descension of the curve occurs. FTIR spectra were recorded on a ThermoScientific Nicolet iS50 FTIR spectrometer using an attenuated total reflectance accessory. The transmission mode was used, and the resolution was 16 cm^–1^. SEC measurements were carried out on a Malvern VISCOTEK GPCmax-Viscotek TDA305 with mixed D5000-D3000-D1000-DGuard column and refractive index detector. Column temperature was 55 °C and injection volume was 100 μL. DMF was used as an eluent at a flow rate of 0.7 mL/min and molecular weights were calculated using both poly(methyl methacrylate) and poly(2-ethyl-2-oxazoline) (PEOZ) standards developed in our group.^25^

## Supporting Information Available

Synthesis of monomers and copolymers (Section 1) and the curing data of TLCs (Section 2) (PDF)
