# Supplementary material for: Amphiphilic Polyoxazoline Copolymer–Imidazole Complexes as Tailorable Thermal Latent Curing Agents for One-Component Epoxy Resins
Source: ACS Omega. 2023 Nov 30;8(49):47173–86. doi: 10.1021/acsomega.3c07177 (PMC10720278; doi:10.1021/acsomega.3c07177)
Supplement: Supplementary file 1 — ao3c07177_si_001.pdf [file ao3c07177_si_001.pdf]

Amphiphilic Polyoxazoline Copolymer-Imidazole Complexes as Tailorable Thermal Latent Curing Agents for One-Component Epoxy Resins

Taha Behroozi Kohlan[1, 2, ‡], Asu Ece Atespare[1, 2], Mehmet Yildiz[1,2], Yusuf Ziya Menceloglu[1, 2], Serkan Unal[1, 2], Bekir Dizman[1, 2] *

[1] Integrated Manufacturing Technologies Research and Application Center & Composite Technologies Center of Excellence, Sabanci University, Istanbul, Turkey

[2] Faculty of Engineering and Natural Sciences, Materials Science and Nano Engineering, Sabanci University, Istanbul, Turkey

*Corresponding author: Bekir Dizman

1. Synthesis of Monomers and Copolymers

Detailed procedures for the synthesis, purification, and characterization of the monomers and copolymers can be found in the publication by our group on the synthesis of these materials[1].

1.1. Synthesis of Monomers

PrOZ was synthesized by reacting butyronitrile (60 g, 0.87 mol, 1 equiv) as the starting material with ethanol amine (58.5 g, 0.95 mol, 1.1 equiv) and $Zn(OAc)_2.2H_2O$ (3.82 g, 0.017 mol, 0.02 equiv) in a single step reaction. The reaction mixture was heated to 130 °C to reflux under N_2 overnight. After completion of the reaction, the crude solution was diluted with DCM and washed with sodium bicarbonate solution twice. The organic phase was dried over sodium sulfate followed by its filtration and evaporation of DCM. The colorless liquid monomer was obtained after distillation (52g, yield: 53%).

In contrast to PrOZ, PeOZ was synthesized in three steps using hexanoic acid as the starting material. In the first step, hexanoyl chloride was obtained by reacting ice-cooled hexanoic acid (50 g, 0.43 mol, 1 eq) with oxalyl chloride (82 g, 0.64 mol, 1.5 eq) added dropwise under N_2 atmosphere. The reaction was left to warm to room temperature and was refluxed for 1 h. The excess amount of oxalyl chloride was then removed by distillation from the reaction crude. In the second step, 2-chloroethylamine hydrochloride (62.3 g, 0.537 mol, 1.25 eq), triethylamine (161.8 mL, 1.161 mol, 2.7 eq), and DCM was mixed in a flask and cooled to 0 °C. Then, hexanoyl chloride was added dropwise under N_2 atmosphere. The reaction mixture was left to warm to room temperature and kept stirring overnight. The reaction mixture was filtered and the filtrate was concentrated under reduced pressure. Obtained solids were dissolved in DCM and washed twice with sodium bicarbonate and once with brine. The organic phase was dried over sodium sulfate followed by its filtration. The product was obtained after the evaporation of DCM. To carry out

‡ Current Address: Department of Fibre and Polymer Technology, KTH Royal Institute of Technology, Stockholm, Sweden

the ring closure step, obtained N-(2-chloroethyl) hexanamide (1 eq) was dissolved in methanol to form 10 wt/v% solution and added to a methanolic solution of NaOH (1.5 eq). The solution was heated to reflux overnight. To purify, the reaction mixture was filtered and the filtrate was concentrated under reduced pressure. Later, it was diluted with DI water and the product was extracted to DCM. The obtained organic phase was dried over sodium sulfate, filtered, and dried under reduced pressure. The monomer was obtained by distillation at 38 °C and 1 mbar as a colorless liquid (24g, yield: 54%).

1.2. Purification of copolymers

Purification of PEOZ-PPrOZ 1K and PEOZ-PPeOZ 1K copolymers

To purify PEOZ-PPrOZ 1K and PEOZ-PPeOZ 1K copolymers of any composition, after the completion of the termination step of the polymerization, the reaction solution was evaporated. Obtained solids were mixed with DI water followed by evaporation of water to remove chlorobenzene azeotropically. This step was performed twice for all copolymers regardless of their solubility or insolubility in water. Later, the obtained solids were dissolved in DCM and filtered to remove excess KOH. Then, the organic phase was dried and DCM was evaporated. The obtained polymers were dissolved in methanol, precipitated in ice cooled diethyl ether, filtered, and dried under vacuum.

Purification of PEOZ-PPhOZ 1K copolymers

PEOZ-PPhOZ 1K copolymers of any composition were purified following a similar procedure reported above. After the termination step, the reaction mixture was evaporated and to remove chlorobenzene, water was added to the obtained solids followed by its evaporation. Later, the obtained solids were dissolved in THF and filtered to remove excess KOH. THF was evaporated and the obtained copolymer was dissolved in methanol, precipitated in diethyl ether, filtered, and dried under vacuum.

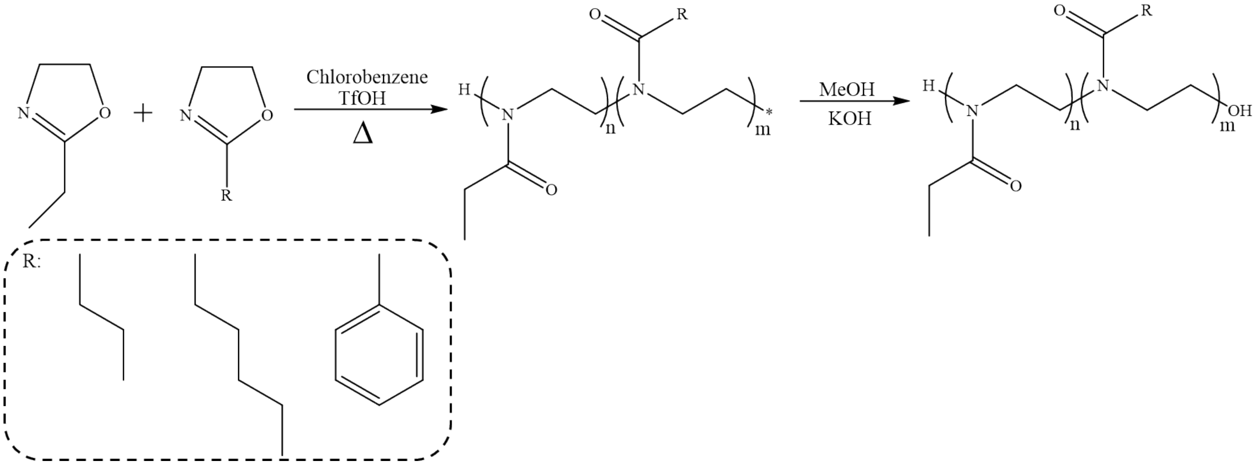

2. Curing studies of prepared one-component systems

2.1. Curing data for PEOZ-PPrOZ 50:50 1K-based systems

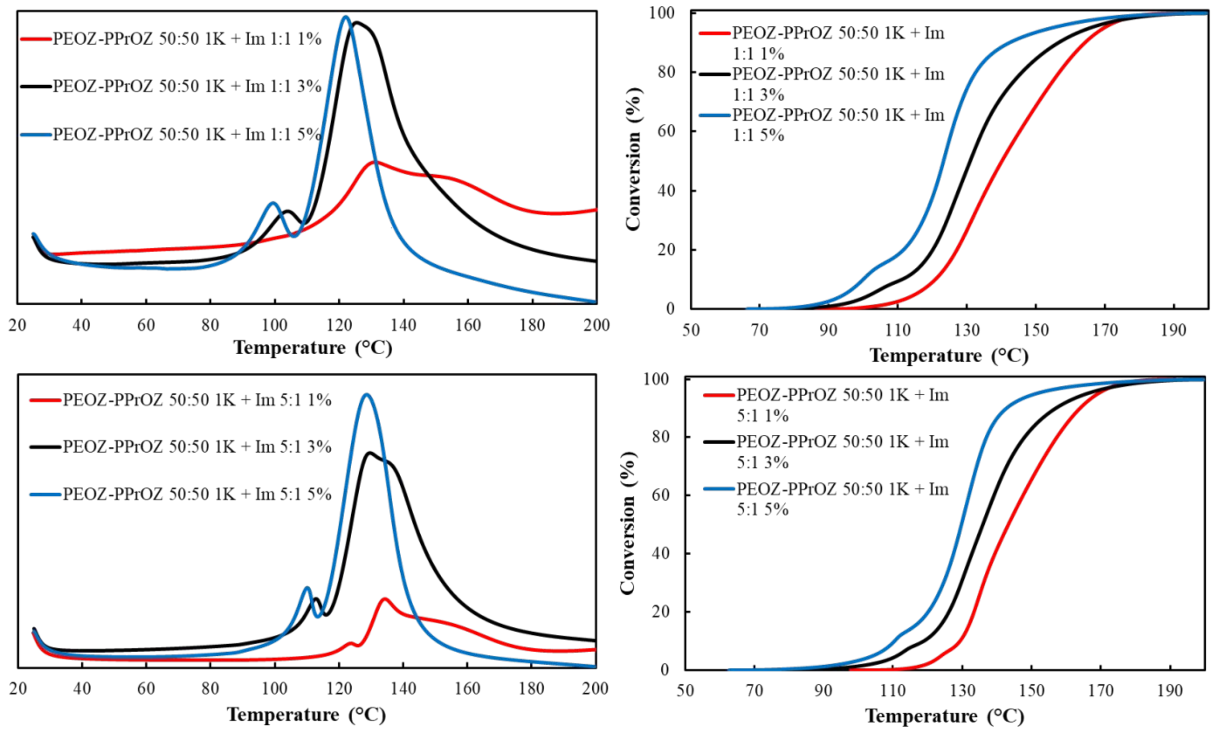

Figure S1. DSC thermograms of curing DGEBA with PEOZ-PPrOZ 50:50 1K based systems and their conversion curves.

Table S1. Dynamic DSC data of PEOZ-PPrOZ 50:50 1K based systems at different copolymer: Im and Im: DGEBA ratios.

Copolymer:Im ratio	Im:DGEBA ratio (wt%)	Normalized enthalpy (J/g)	1st peak onset (°C)	2nd peak onset (°C)	Main peak temp. (°C)	Left limit (°C)
1:1	1%	184.85	-	114.36	130.91	86.17
1:1	3%	524.70	93.62	111.11	125.42	66.89
1:1	5%	431.25	90.41	108.19	122.15	66.37
5:1	1%	129.38	116.56	126.44	134.27	97.84
5:1	3%	483.75	105.63	117.49	129.52	69.04
5:1	5%	494.40	103.45	114.69	128.58	62.75

2.2. Curing data for PEOZ-PPrOZ 75:25 1K-based systems

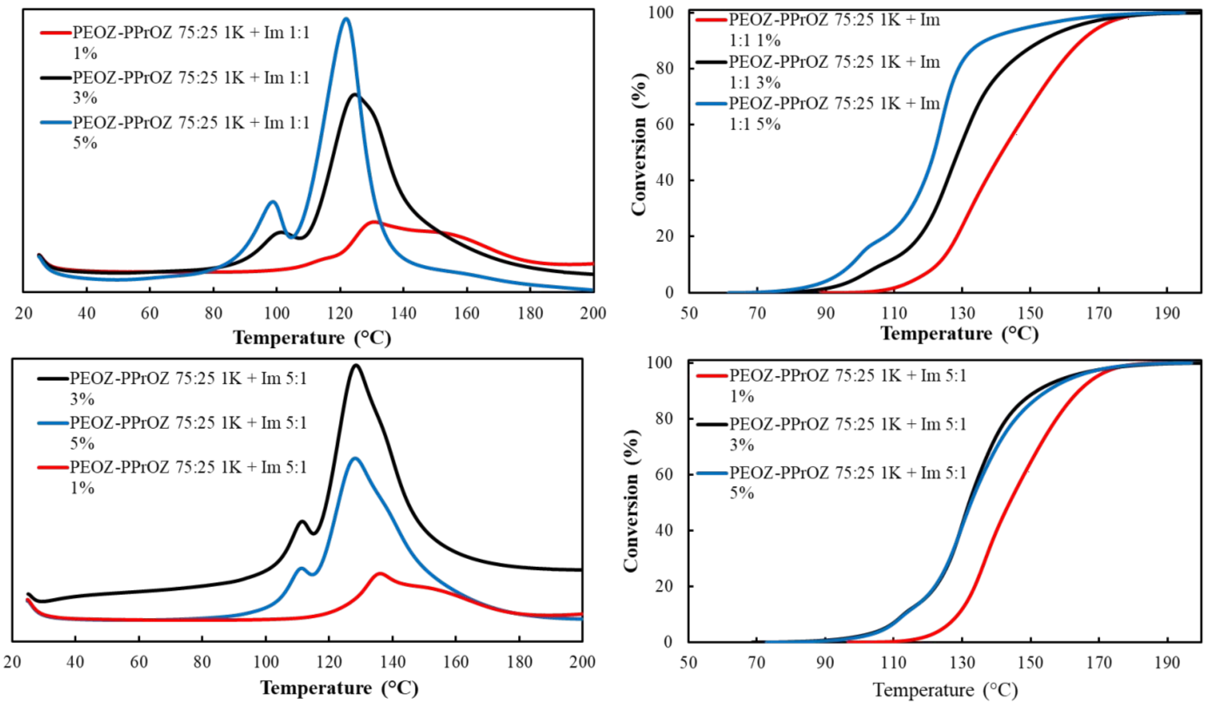

Figure S2. DSC thermograms of curing DGEBA with PEOZ-PPrOZ 75:25 1K-based systems and their conversion curves.

Table S2. Dynamic DSC data of PEOZ-PPrOZ 75:25 1K based systems at different copolymer: Im and Im: DGEBA ratios.

Copolymer:Im ratio	Im:DGEBA ratio (wt%)	Normalized enthalpy (J/g)	1st peak onset (°C)	2nd peak onset (°C)	Main peak temp. (°C)	Left limit (°C)
1:1	1%	168.38	106.68	117.45	130.68	88.72
1:1	3%	500.66	89.49	109.40	124.60	66.44
1:1	5%	534.50	88.32	106.95	121.96	61.55
5:1	1%	101.02	-	124.70	136.11	96.64
5:1	3%	433.67	103.60	115.90	128.36	68.63
5:1	5%	364.22	102.85	114.81	128.23	73.07

2.3. Curing data for PEOZ-PPeOZ 25:75 1K-based systems

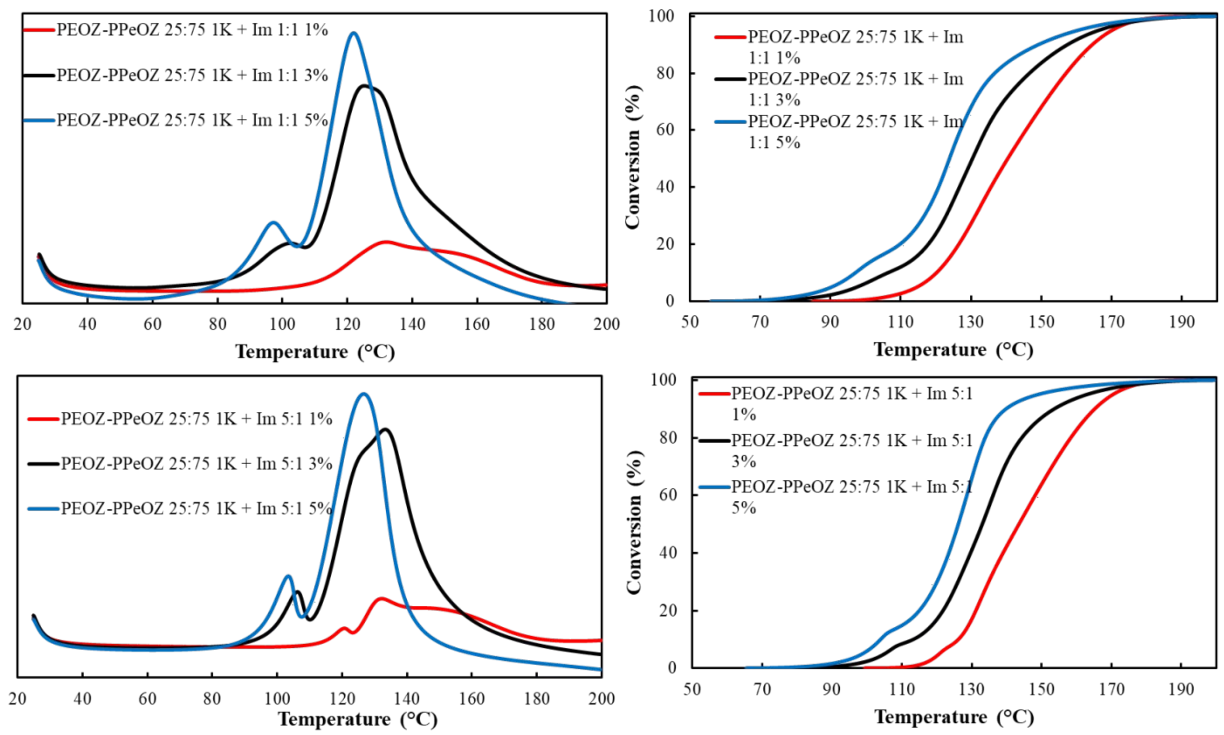

Figure S3. DSC thermograms of curing DGEBA with PEOZ-PPeOZ 25:75 1K based systems and their conversion curves.

Table S3. Dynamic DSC data of PEOZ-PPeOZ 25:75 1K-based systems at different copolymer: Im and Im: DGEBA ratios.

Copolymer:Im ratio	Im:DGEBA ratio (wt%)	Normalized enthalpy (J/g)	1st peak onset (°C)	2nd peak onset (°C)	Main peak temp. (°C)	Left limit (°C)
1:1	1%	135.46	-	110.15	131.90	84.91
1:1	3%	498.30	87.53	108.59	125.26	58.07
1:1	5%	558.97	85.55	105.97	121.92	56.04
5:1	1%	117.16	114.32	122.65	132.11	99.34
5:1	3%	491.02	97.66	111.70	133.41	70.80
5:1	5%	460.06	94.55	126.71	126.71	65.50

2.4. Curing data for PEOZ-PPeOZ 50:50 1K-based systems

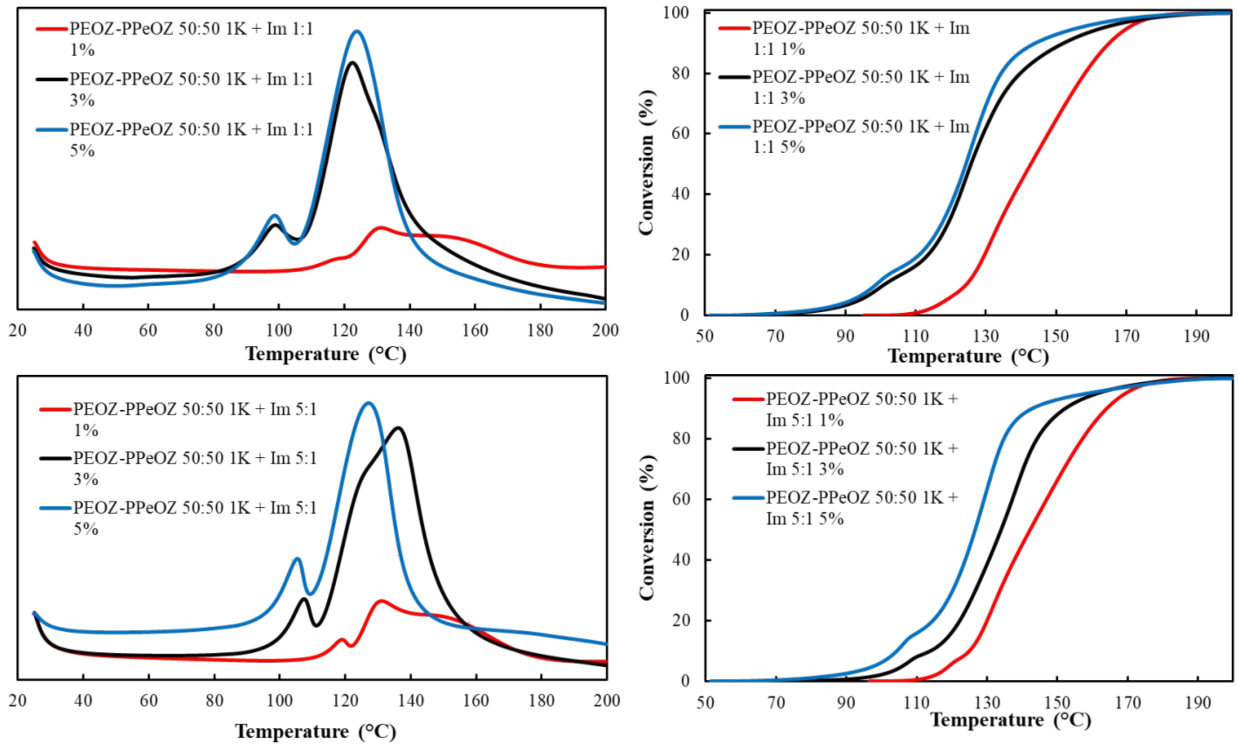

Figure S4. DSC thermograms of curing DGEBA with PEOZ-PPeOZ 50:50 1K based systems and their conversion curves.

Table S4. Dynamic DSC data of PEOZ-PPeOZ 50:50 1K based systems at different copolymer: Im and Im: DGEBA ratios.

Copolymer:Im ratio	Im:DGEBA ratio (wt%)	Normalized enthalpy (J/g)	1st peak onset (°C)	2nd peak onset (°C)	Main peak temp. (°C)	Left limit (°C)
1:1	1%	116.40	108.45	119.46	131.12	95.15
1:1	3%	498.71	88.54	107.29	122.59	55.03
1:1	5%	537.91	88.52	105.71	123.75	51.28
5:1	1%	145.99	112.81	121.27	131.06	96.57
5:1	3%	490.87	99.81	111.54	136.09	59.71
5:1	5%	410.35	98.05	109.95	127.16	51.46

2.5. Curing data for PEOZ-PPeOZ 75:25 1K-based systems

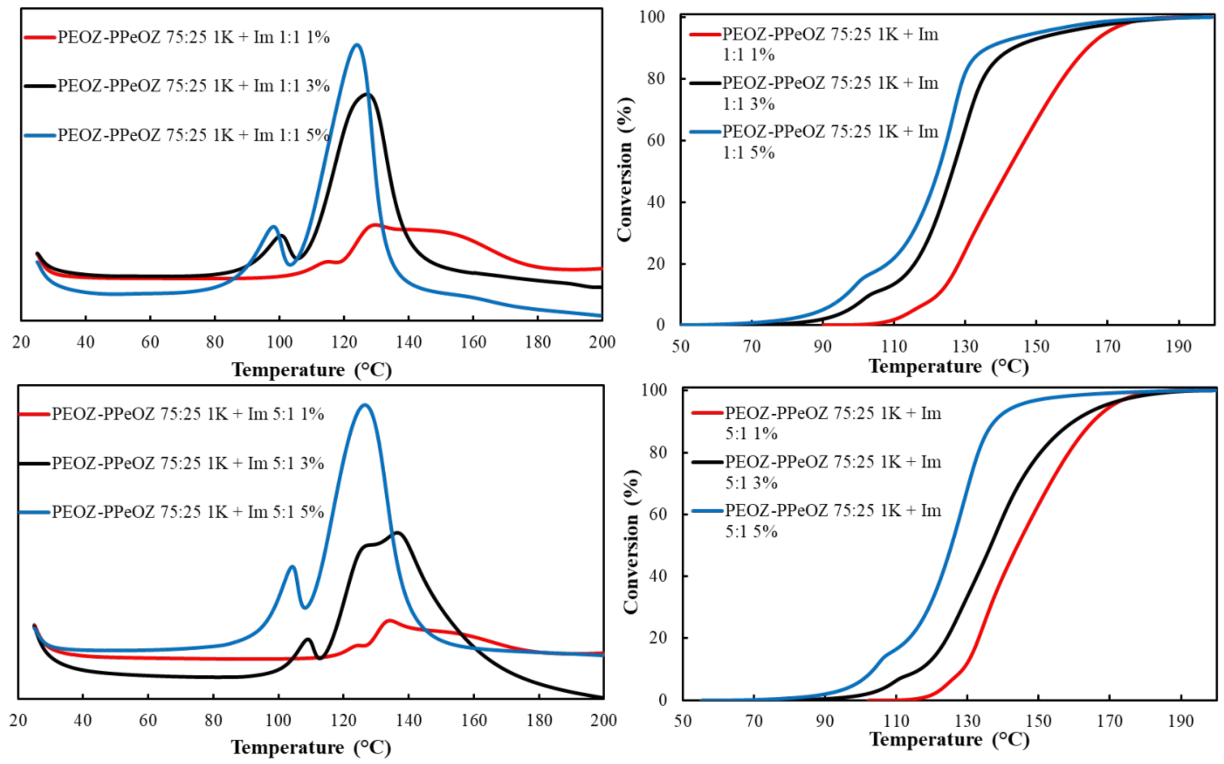

Figure S5. DSC thermograms of curing DGEBA with PEOZ-PPeOZ 75:25 1K based systems and their conversion curves.

Table S5. Dynamic DSC data of PEOZ-PPeOZ 75:25 1K based systems at different copolymer: Im and Im: DGEBA ratios.

Copolymer:Im ratio	Im:DGEBA ratio (wt%)	Normalized enthalpy (J/g)	1st peak onset (°C)	2nd peak onset (°C)	Main peak temp. (°C)	Left limit (°C)
1:1	1%	185.33	106.16	117.59	129.67	90.25
1:1	3%	428.51	90.90	108.58	127.19	55.78
1:1	5%	543.01	86.79	105.07	123.99	48.55
5:1	1%	88.65	117.71	125.65	134.17	102.29
5:1	3%	443.51	102.12	113.42	136.55	67.40
5:1	5%	476.86	96.31	108.74	126.47	55.30

2.6. Curing data for PEOZ-PPhOZ 25:75 1K-based systems

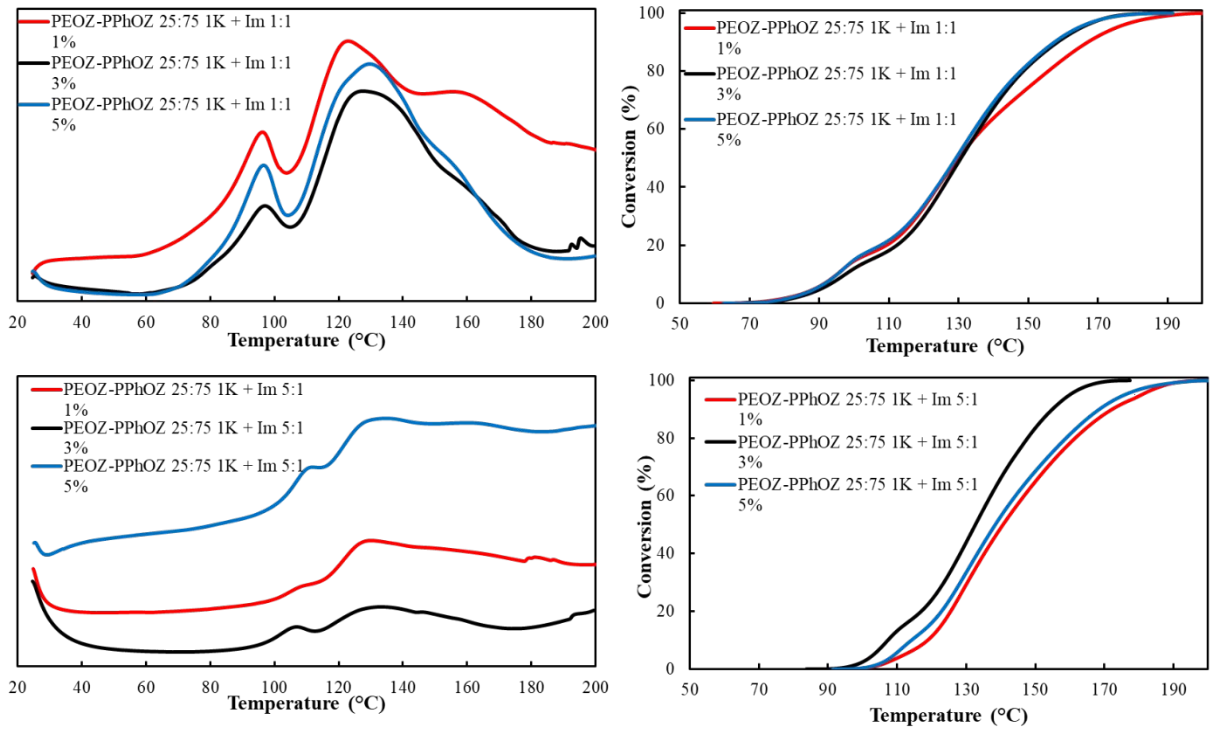

Figure S6. DSC thermograms of curing DGEBA with PEOZ-PPhOZ 25:75 1K-based systems and their conversion curves.

Table S6. Dynamic DSC data of PEOZ-PPhOZ 25:75 1K based systems at different copolymer: Im and Im: DGEBA ratios.

Copolymer: Im ratio	Im: DGEBA ratio (wt%)	Normalized enthalpy (J/g)	1st peak onset (°C)	2nd peak onset (°C)	Main peak temp. (°C)	Left limit (°C)
1:1	1%	270.01	87.27	103.02	122.29	59.57
1:1	3%	263.71	87.24	103.79	126.77	62.90
1:1	5%	324.66	84.99	101.89	129.40	62.44
5:1	1%	79.22	-	111.98	129.03	89.93
5:1	3%	46.14	99.31	108.13	131.72	83.66
5:1	5%	105.68	91.00	109.73	129.88	91.30

2.7. Curing data for PEOZ-PPhOZ 50:50 1K-based systems

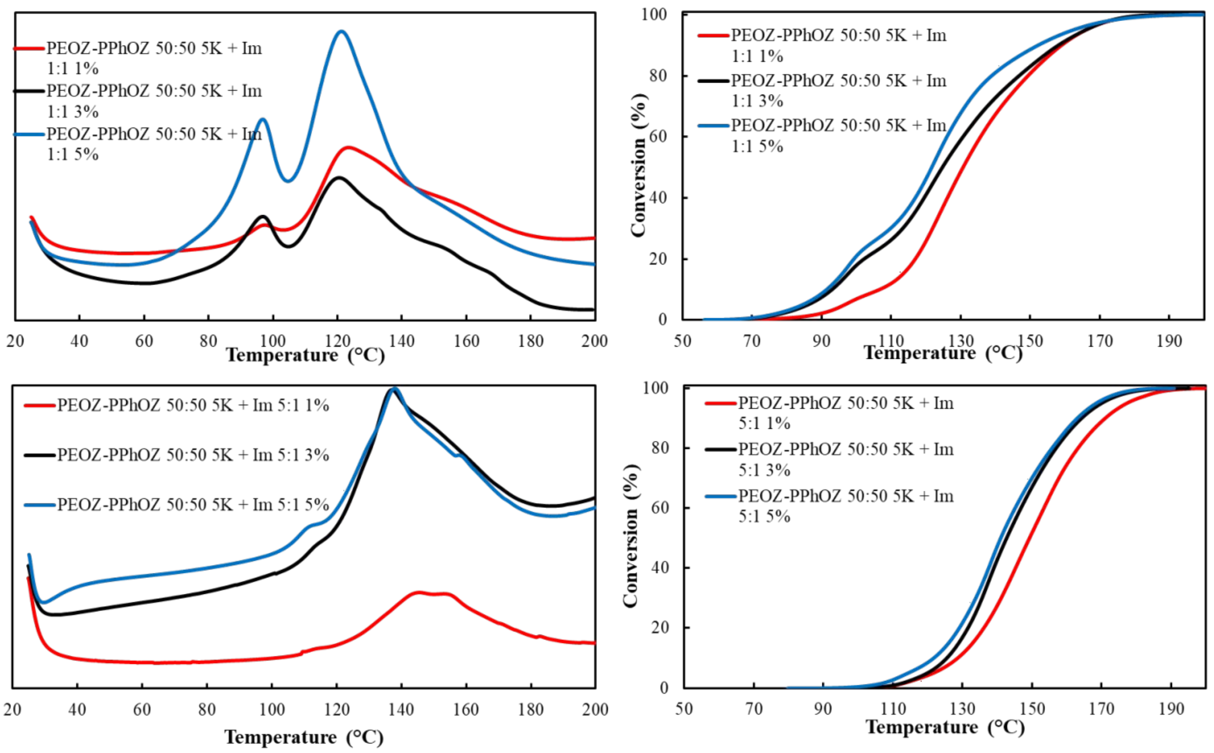

Figure S7. DSC thermograms of curing DGEBA with PEOZ-PPhOZ 50:50 1K based systems and their conversion curves.

Table S7. Dynamic DSC data of PEOZ-PPhOZ 50:50 1K based systems at different copolymer: Im and Im: DGEBA ratios.

**Copolymer: Im ratio**	**Im: DGEBA ratio (wt%)**	**Normalized enthalpy (J/g)**	**1st peak onset (°C)**	**2nd peak onset (°C)**	**Main peak temp. (°C)**	**Left limit (°C)**
1:1	1%	188.86	89.69	106.29	123.30	63.10
1:1	3%	273.35	86.10	101.74	120.58	61.36
1:1	5%	452.43	86.14	102.51	121.12	56.28
5:1	1%	44.40	-	120.76	145.34	90.92
5:1	3%	100.04	105.31	121.35	137.00	88.03
5:1	5%	97.18	101.76	115.11	137.93	79.86

2.8. Curing data for PEOZ-PPhOZ 75:25 1K based systems

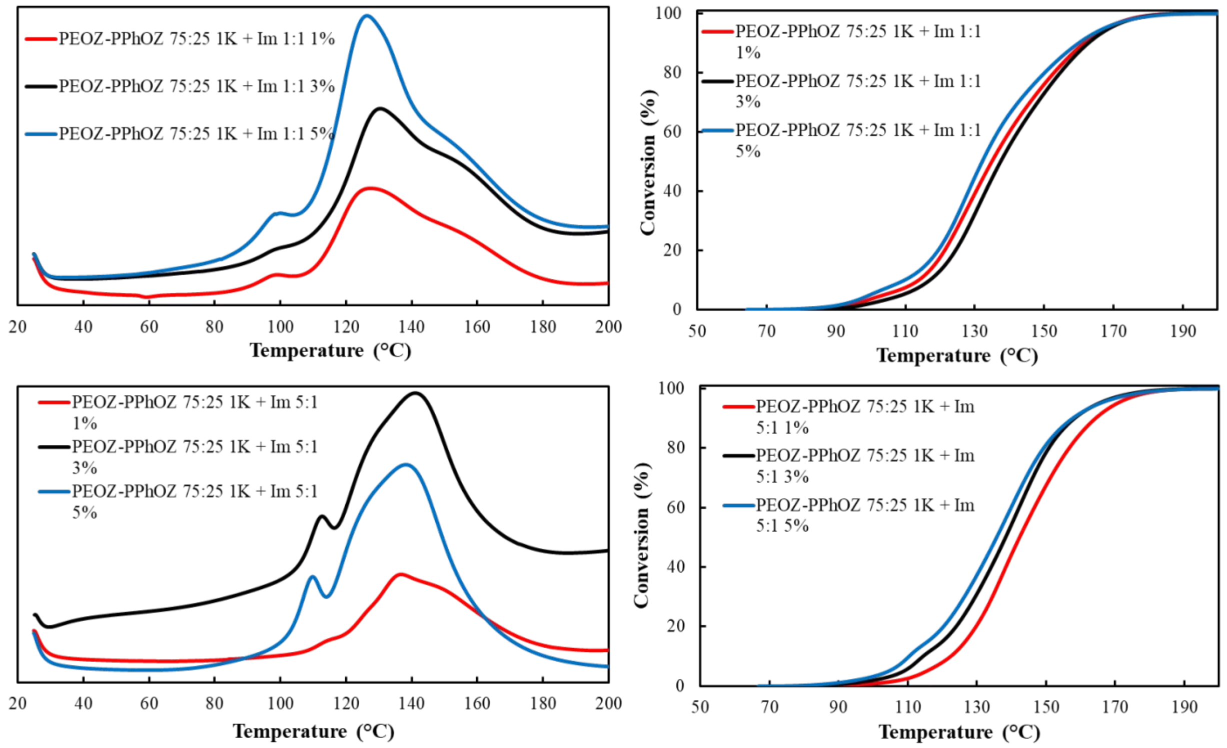

Figure S8. DSC thermograms of curing DGEBA with PEOZ-PPhOZ 75:25 1K based systems and their conversion curves.

Table S8. Dynamic DSC data of PEOZ-PPhOZ 75:25 1K based systems at different copolymer: Im and Im: DGEBA ratios.

**Copolymer: Im ratio**	**Im: DGEBA ratio (wt%)**	**Normalized enthalpy (J/g)**	**1st peak onset (°C)**	**2nd peak onset (°C)**	**Main peak temp. (°C)**	**Left limit (°C)**
1:1	1%	165.11	87.28	108.95	127.18	71.66
1:1	3%	220.55	87.76	112.10	130.35	76.13
1:1	5%	336.71	87.00	109.36	126.29	64.25
5:1	1%	138.79	106.60	117.68	136.63	77.78
5:1	3%	317.19	105.13	112.47	140.72	71.97
5:1	5%	376.85	102.14	109.21	138.23	66.92

Conversion curves for effects of composition

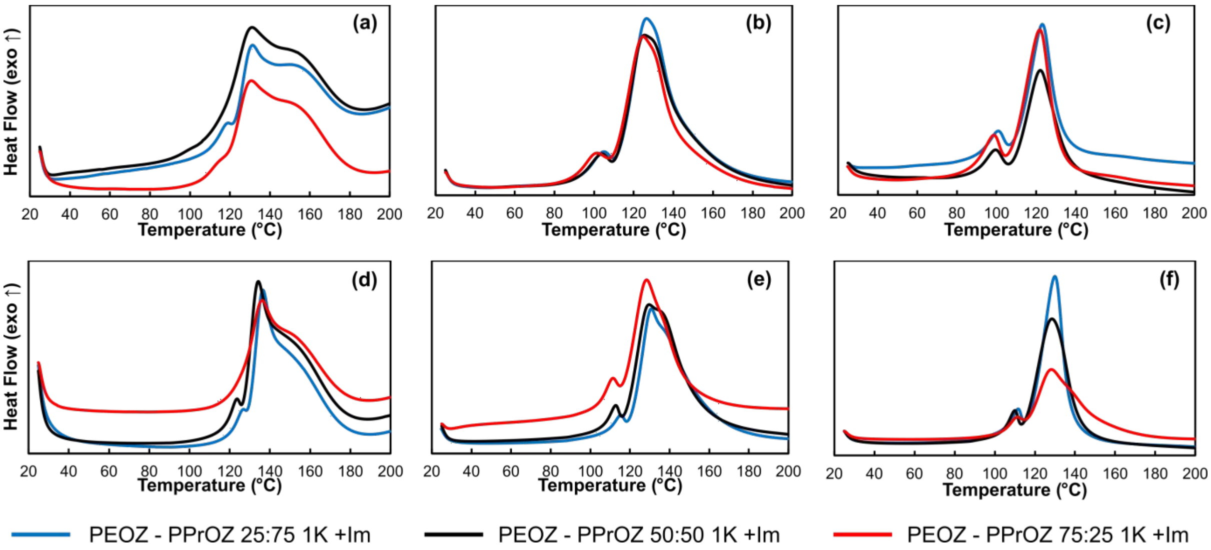

Figure S9. DSC thermograms of curing DGEBA with PEOZ-PPrOZ-based TLCs at 1:1, 1 wt % (a), 1:1, 3 wt % (b), 1:1, 5 wt% (c), 5:1, 1 wt % (d), 5:1, 3 wt % (e), and 5:1 5 wt % (f). Heating rate: 10 °C/min.

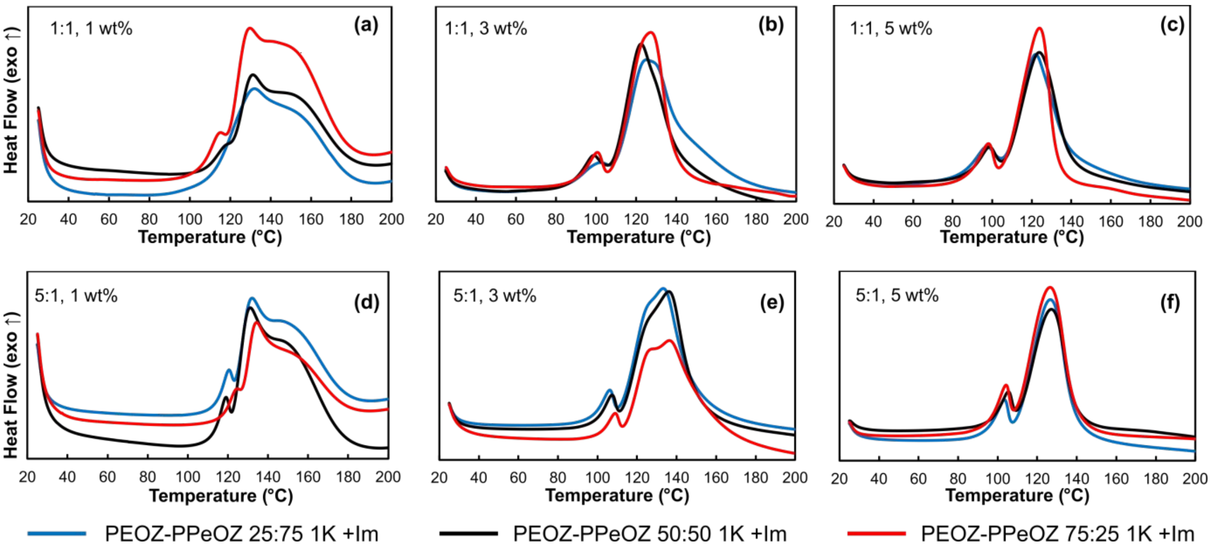

Figure S10. DSC thermograms and conversion curves of curing DGEBA with PEOZ-PPeOZ and Im complexes at 1:1, 1 wt % (a), 1:1, 3 wt % (b), 1:1, 5 wt% (c), 5:1, 1 wt % (d), 5:1, 3 wt % (e), and 5:1 5 wt % (f). Heating rate: 10 °C/min.

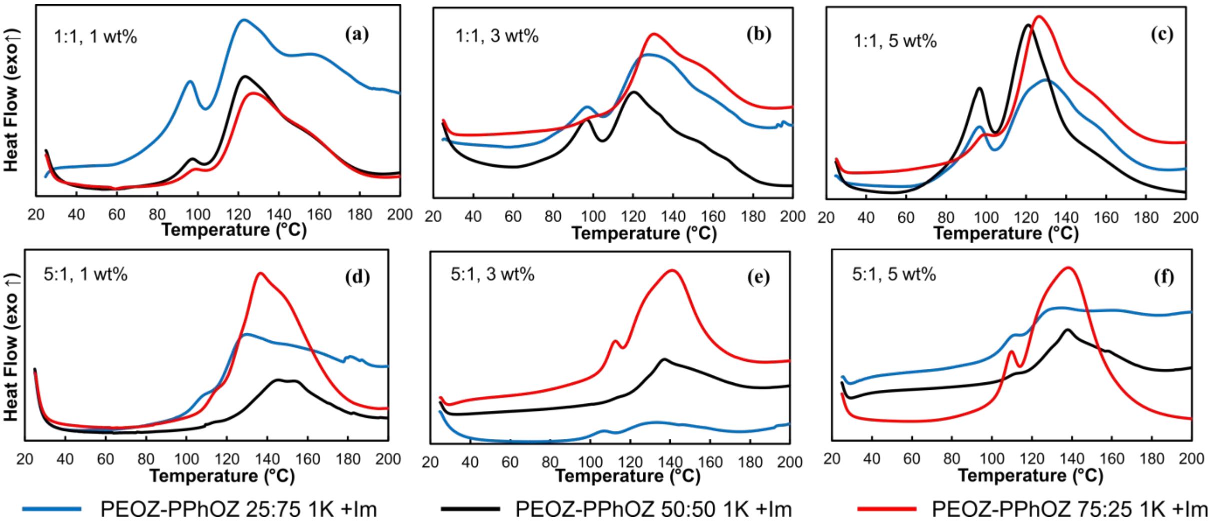

Figure S11. DSC thermograms and conversion curves of curing DGEBA with PEOZ-PPhOZ and Im complexes at 1:1, 1 wt % (a), 1:1, 3 wt % (b), 1:1, 5 wt% (c), 5:1, 1 wt % (d), 5:1, 3 wt % (e), and 5:1 5 wt % (f). Heating rate: 10 °C/min.

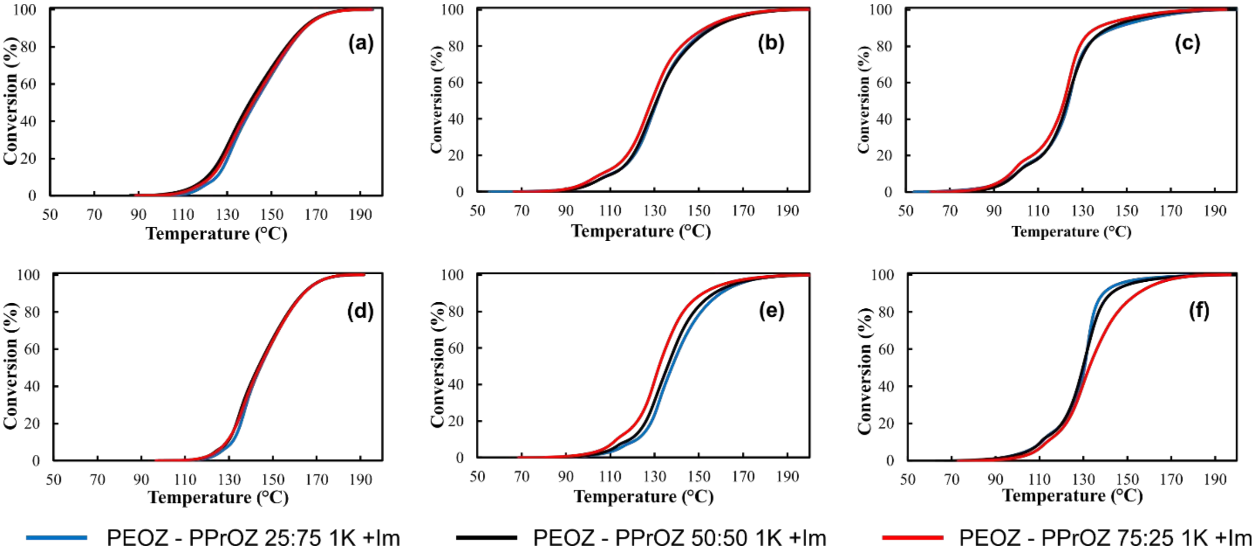

Figure S12. Conversion curves of curing DGEBA with PEOZ-PPrOZ based TLCs at 1:1, 1 wt % (a), 1:1, 3 wt % (b), 1:1, 5 wt% (c), 5:1, 1 wt % (d), 5:1, 3 wt % (e), and 5:1 5 wt % (f).

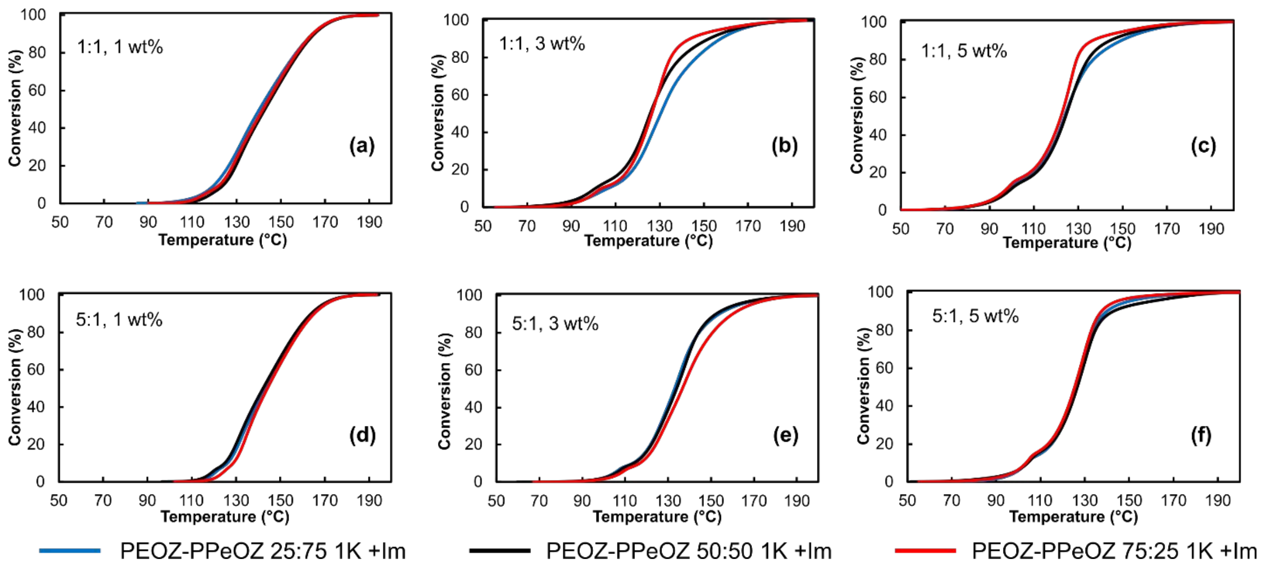

Figure S13. Conversion curves of curing DGEBA with PEOZ-PPeOZ and Im complexes at 1:1, 1 wt % (a), 1:1, 3 wt % (b), 1:1, 5 wt% (c), 5:1, 1 wt % (d), 5:1, 3 wt % (e), and 5:1 5 wt % (f).

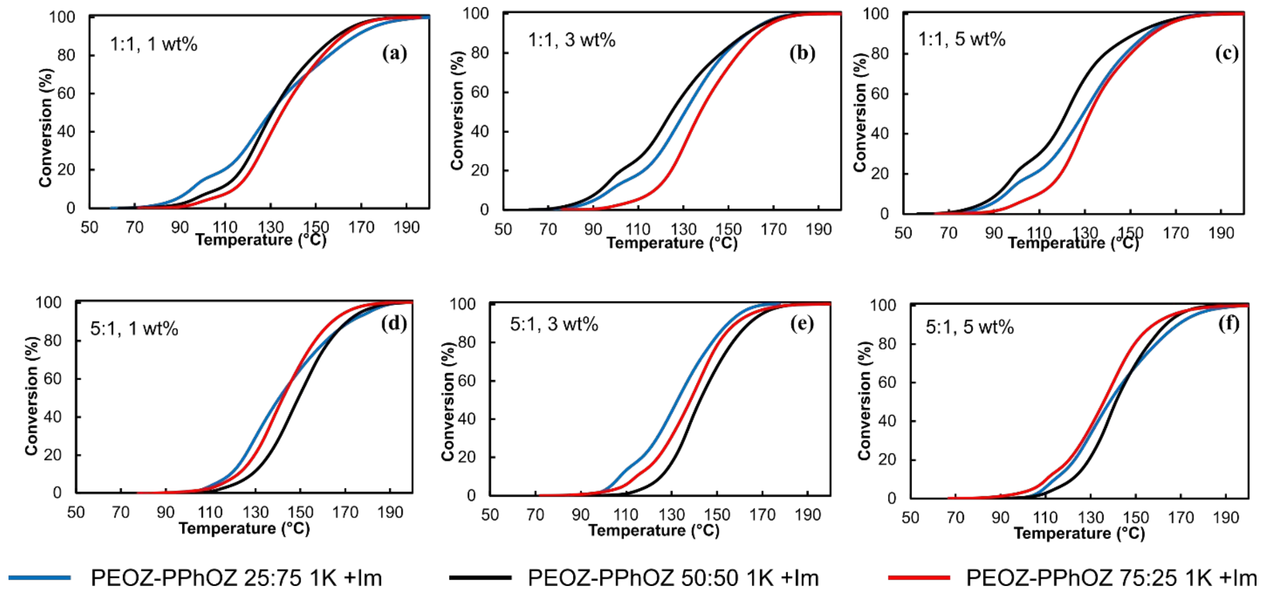

Figure S14. conversion curves of curing DGEBA with PEOZ-PPhOZ and Im complexes at 1:1, 1 wt % (a), 1:1, 3 wt % (b), 1:1, 5 wt% (c), 5:1, 1 wt % (d), 5:1, 3 wt % (e), and 5:1 5 wt % (f).

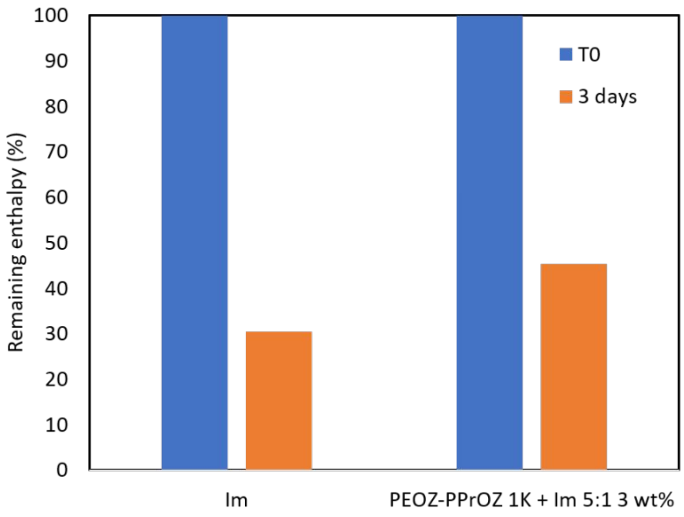

Figure S15. Stability of prepared OCER after 3 days of storage.

References

(1) Kohlan, T. B.; Atespare, A. E.; Yildiz, M.; Menceloglu, Y. Z.; Unal, S.; Dizman, B. Synthesis and Structure–Property Relationship of Amphiphilic Poly(2-Ethyl-Co-2-(Alkyl/Aryl)-2-Oxazoline) Copolymers. *ACS Omega* **2022**. https://doi.org/10.1021/ACSOMEGA.2C04809.
